# Plants Encode a General siRNA Suppressor That Is Induced and Suppressed by Viruses

**DOI:** 10.1371/journal.pbio.1002326

**Published:** 2015-12-22

**Authors:** Nahid Shamandi, Matthias Zytnicki, Cyril Charbonnel, Emilie Elvira-Matelot, Aurore Bochnakian, Pascale Comella, Allison C. Mallory, Gersende Lepère, Julio Sáez-Vásquez, Hervé Vaucheret

**Affiliations:** 1 Institut Jean-Pierre Bourgin, UMR 1318, INRA AgroParisTech CNRS, Université Paris-Saclay, Versailles, France; 2 Université Paris-Sud, Université Paris-Saclay, Orsay, France; 3 URGI, INRA, Versailles, France; 4 CNRS, UMR 5096, LGDP, Perpignan, France; 5 Université Perpignan Via Domitia, UMR 5096, LGDP, Perpignan, France; Cold Spring Harbor Laboratory, UNITED STATES

## Abstract

Small RNAs play essential regulatory roles in genome stability, development, and responses to biotic and abiotic stresses in most eukaryotes. In plants, the RNaseIII enzyme DICER-LIKE1 (DCL1) produces miRNAs, whereas DCL2, DCL3, and DCL4 produce various size classes of siRNAs. Plants also encode RNASE THREE-LIKE (RTL) enzymes that lack DCL-specific domains and whose function is largely unknown. We found that virus infection induces RTL1 expression, suggesting that this enzyme could play a role in plant–virus interaction. To first investigate the biochemical activity of RTL1 independent of virus infection, small RNAs were sequenced from transgenic plants constitutively expressing RTL1. These plants lacked almost all DCL2-, DCL3-, and DCL4-dependent small RNAs, indicating that RTL1 is a general suppressor of plant siRNA pathways. In vivo and in vitro assays revealed that RTL1 prevents siRNA production by cleaving dsRNA prior to DCL2-, DCL3-, and DCL4-processing. The substrate of RTL1 cleavage is likely long-perfect (or near-perfect) dsRNA, consistent with the RTL1-insensitivity of miRNAs, which derive from DCL1-processing of short-imperfect dsRNA. Virus infection induces *RTL1* mRNA accumulation, but viral proteins that suppress RNA silencing inhibit RTL1 activity, suggesting that RTL1 has evolved as an inducible antiviral defense that could target dsRNA intermediates of viral replication, but that a broad range of viruses counteract RTL1 using the same protein toolbox used to inhibit antiviral RNA silencing. Together, these results reveal yet another level of complexity in the evolutionary battle between viruses and plant defenses.

## Abstract 2

Viral infection of plants triggers the expression of an RNaseIII enzyme that represses the production of siRNAs by cleaving their long dsRNA precursors. Read the accompanying Synopsis.

## Author Summary

Most eukaryotes produce essential regulatory molecules called small RNAs. These molecules are produced primarily by a class of RNaseIII enzymes called DICER, which excises small RNA duplexes from long double-stranded (ds)RNA precursor molecules. Plants also encode several RNaseIII enzymes called RNASE THREE-LIKE (RTL), but the function of these proteins is largely unknown. Here, we show that RTL1 represses small RNA production by cleaving dsRNA before DICER can process them. RTL1 appears to specifically act on the templates of a class of small RNAs called siRNAs, but not on miRNA precursors, suggesting that it cleaves long-perfect (or near-perfect) dsRNA, but not short-imperfect dsRNA. We also found that RTL1 expression is induced after virus infection, suggesting that RTL1 could act as an inducible antiviral defense by destroying dsRNA intermediates of viral replication. Our findings suggest that viruses have evolved to inhibit RTL1 activity, ultimately resulting in successful viral infection.

## Introduction

In eukaryotes, the biogenesis of small RNAs is either Dicer-dependent or Dicer-independent. Dicer-independent small RNAs resulting from the action of RNA-dependent RNA polymerases, exoribonucleases, Argonaute (AGO) proteins, or a combination of these factors have been found in fungi, invertebrates, and mammals but not in plants or protists [1–4]. In contrast, Dicer-dependent small RNAs are found in every eukaryotic kingdom [5,6], with the notable exception of a few yeast species [7–9]. Dicer enzymes belong to the family of RNaseIII proteins, which are double-stranded (ds)RNA-specific endonucleases. All members of the RNaseIII family contain a characteristic RNaseIII domain composed of a highly conserved stretch of nine amino acid residues known as the RNaseIII signature motif [10]. RNaseIII proteins vary widely in length, from 200 to 2,000 amino acids, and have been subdivided into four classes based on their domain composition [11]. Class I is the simplest and the smallest, containing a single RNaseIII domain and a dsRNA (double stranded RNA) binding domain (DRB); the bacterial and bacteriophage RNaseIII proteins belong to this class. Class II proteins, like class I, contain both an RNaseIII domain and a DRB but are distinguished from class I by the presence of a highly variable N-terminal domain extension and include the *Saccharomyces cerevisiae* Rnt1 and *S*. *pombe* Pac1 proteins. Both of these yeast proteins are longer than bacterial RNaseIII proteins and contain an additional 100 amino acids at their N-terminus. Class III proteins have a DRB and two RNaseIII domains and include Drosha, which is involved in the first cut of miRNA precursors in animals but is incapable of producing small RNAs by itself. Class IV proteins correspond to animal and plant Dicer and contain an RNA helicase domain, a PAZ domain, either one or two RNaseIII domains, and one or two DRB domains. Animal and plant Dicer proteins are the only RNaseIII proteins that have been shown to produce small RNAs in the size range of 18–24 nt, with the exception of class II RNaseIII from a few budding yeasts [9].

The plant model *Arabidopsis* encodes four Dicer-like (DCL) proteins [12], which produce various classes of small RNAs. DCL1 produces the majority of microRNAs (miRNAs), which average around 21 nt in length and derive from relatively short, imperfectly double-stranded stem-loop RNA precursors transcribed from nonprotein coding *MIR* genes. By contrast, DCL2, DCL3, and DCL4 produce 22, 24, and 21 nt small interfering RNAs (siRNAs), respectively, which derive from long, dsRNA precursors originating from either long inverted repeats or from the action of RNA-dependent RNA polymerases (RDR) on single-stranded RNA precursor. Multiple classes of siRNAs exist in plants. The largest class corresponds to POLYMERASE IV-dependent siRNA (p4-siRNAs), which are RDR2- and DCL3-dependent 24 nt siRNAs deriving from transposons and repeats. Trans acting siRNAs (ta-siRNAs) are RDR6- and DCL4-dependent 21-nt siRNAs, which derive from non-protein coding *TAS* genes. Endogenous inverted repeat-derived siRNAs (endoIR-siRNAs) do not depend on an RDR for their production because they derive from very long stem-loops. They come in different flavors, including DCL4-dependent 21 nt, DCL2-dependent 22 nt, and DCL3-dependent 24 nt siRNAs. At last, young miRNAs define a category of small RNA that is intermediate between DCL2-, DCL3-, and DCL4-dependent endoIR-siRNAs and DCL1-dependent old (conserved) miRNAs. Indeed, young miRNAs derive from foldback stem-loops of intermediate sizes, which are mainly processed by DCL4. Most 21 and 22 nt small RNAs are loaded onto AGO1 to guide the cleavage of complementary mRNA [6], whereas 24 nt siRNAs associate with AGO4, which recruits PolV and the chromatin remodeling protein DRD1, leading to transcriptional silencing through histone modification, DNA methylation, and chromatin remodeling [13].

DCL2, DCL3, and DCL4 also produce siRNAs in response to the entry of exogenous genetic material. For example, transient transgene expression leads to a consistent production of 21, 22, and 24 nt siRNAs, even when the transgene has not been designed to produce an RNA that can fold into a dsRNA structure [14]. Following integration of the transgene into the genome, the production of siRNA continues if the transgene produces a dsRNA, but not if it produces a regular single-stranded mRNA. However, in some cases, sense transgenes undergo transcriptional or post-transcriptional silencing for reasons that still remain not perfectly understood. Infection by viruses also leads to a consistent production of 21, 22, and 24 nt siRNAs during the first few days following infection. These siRNAs likely derive from viral RNAs partially folded into dsRNA or from dsRNA intermediates of viral replication. These siRNAs are loaded onto AGO1 and AGO2, which then target single-stranded viral RNA for cleavage [15]. Cleavage products generated by AGO1, but not AGO2 [16], have the capacity to be transformed into dsRNA by RDR1 and RDR6, which are subsequently processed into 21 and 22 nt secondary siRNAs by DCL4 and DCL2, which could in theory reinforce the cleavage of single-stranded viral RNA and thus eliminate the virus. However, this antiviral RNAi mechanism is often counteracted by proteins encoded by the virus itself, which are referred to as viral suppressors of RNA silencing (VSRs). VSRs have been identified in most viruses and target various components of the RNAi machinery, thus blocking RNAi with various efficiencies [17,18]. Because there is generally a positive correlation between the strength of VSRs and the gravity of the symptoms caused by viruses, it has been proposed that the success of infection of a virus depends on the strength of its VSR. However, it remains possible that VSRs affect other function(s) of the plant than simply the RNAi machinery [17].

Aside from DCL1–4, the *Arabidopsis* genome has the capacity to encode five proteins referred to as RNASE THREE-LIKE (RTL). RTL1, RTL2, and RTL3 harbor RNaseIII and DRB domains [19], whereas RTL4 and RTL5 contain only RNaseIII domains [20,21]. RTL1, which carries one RNaseIII domain and one DRB domain, is weakly expressed in roots and below detection level by classical reverse transcriptase polymerase chain reaction (RT-PCR) in other tissues [19]. Mutants defective for RTL1 are not available in any of the public *Arabidopsis* stock centers, and RTL1 function is unknown. RTL2, which contains one RNaseIII domain and two DRB domains, is ubiquitously expressed at low level [19]. RTL2 processes, both in vivo and in vitro, the 3’External Transcribed Spacer (ETS) from ribosomal *45S* pre-rRNA and enhances the production of exogenous siRNAs when overexpressed [22]; however, mutants defective for RTL2 are viable and do not exhibit obvious developmental defects [19]. RTL3 harbors two RNaseIII domains and three DRB domains, but its expression has not been detected in any tested tissue [19]. RTL4 and RTL5 carry a single RNAseIII domain but lack DRB domains, and both genes are expressed in almost every tissue. Whereas the targets of RTL4 remain unknown, mutants defective in RTL4 are impaired in male and female gametophyte formation [20]. RTL5 shares sequence similarity to maize RNC1, which is required for the splicing of several chloroplast group II introns [21]. Here, we show that RTL1 exhibits RNaseIII activity, and that its overexpression prevents the accumulation of all size classes of siRNAs but does not affect the accumulation of DCL1-dependent conserved miRNAs. Our results suggest that RTL1 cleaves long-perfectly (or near-perfectly) paired dsRNA, thus preventing the processing of these substrates by DCL2, DCL3, and DCL4. RTL1 is induced upon infection with a range of viruses, but the VSRs encoded by these viruses inhibit RTL1 activity, suggesting that RTL1 acts in plant antiviral defense while viruses have evolved counterdefenses that inhibit both RTL1 and RNA silencing defenses.

## Results

### Viruses Specifically Induce RTL1

**Fig 1:**
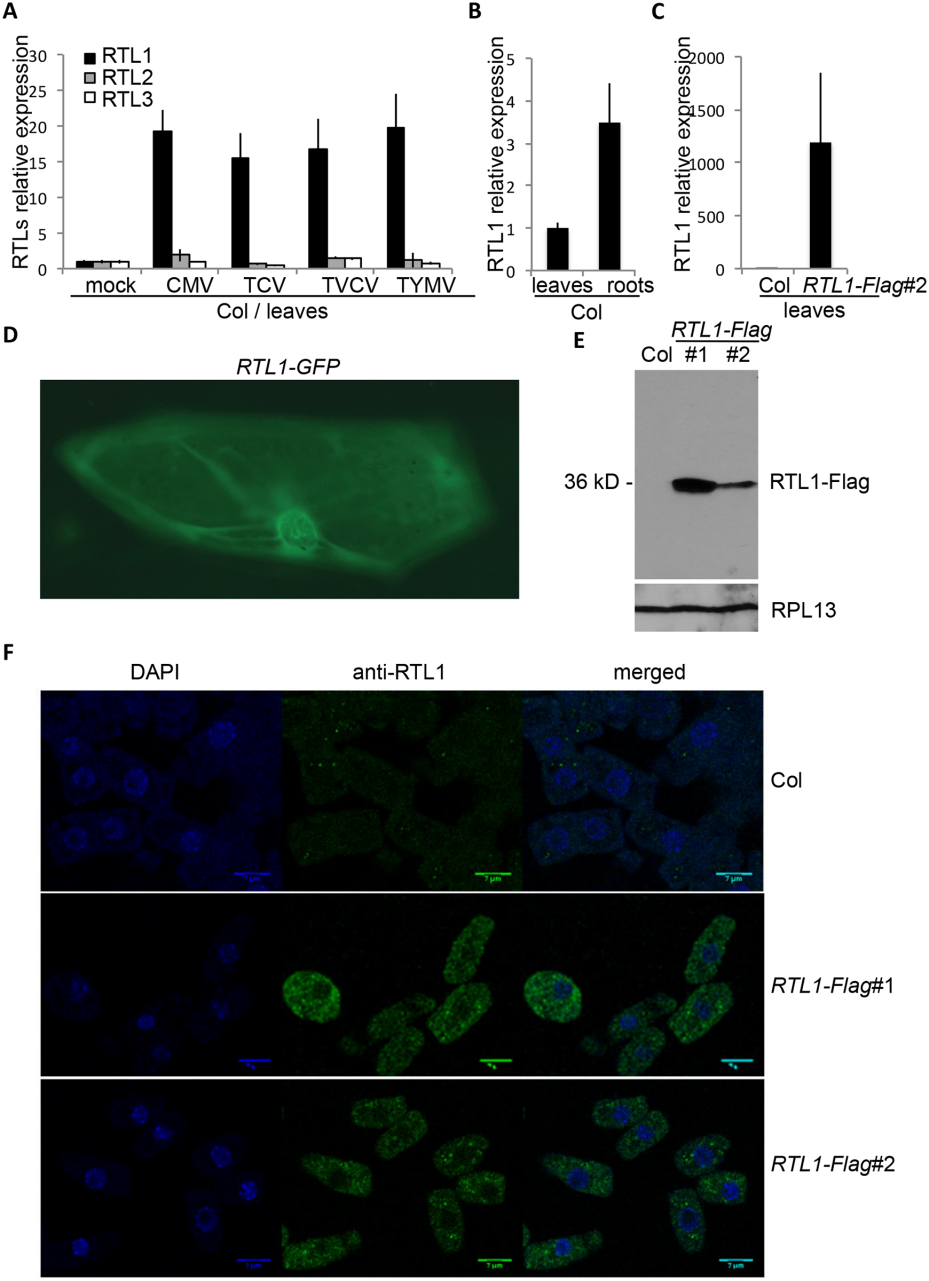
RTL1 expression and localization. **A)** RNA extracted from the total aerial part of wild-type plants (Col) three weeks after inoculation with water (mock), TCV, TVCV, CMV, or TYMV were subjected to oligo-dT reverse transcription followed by qPCR with primers specific to *RTL1*, *RTL2*, or *RTL3*. Analysis was done in triplicate. Results were normalized to *GAPDH*. **B)** RNA extracted from leaves and roots of 3 wk-old wild-type plants (Col) were subjected to oligo-dT reverse transcription followed by qPCR with RTL1 oligos. Analysis was done in triplicate. Results were normalized to *GAPDH*. **C)** RNA extracted from leaves of 3 wk-old wild-type plants (Col) and *35S*:*RTL1-Flag#2* (*RTL1-Flag* #2) plants were subjected to oligo-dT reverse transcription followed by qPCR with RTL1 oligos. Analysis was done in triplicate. Results were normalized to *GAPDH*. **D**) Onion epidermial cells transformed with a *35S*:*RTL1-GFP* construct were imaged using a Zeiss Axioskop 2 microscope and recorded using a Leica DC 300 FX digital camera (Leica). **E**) Proteins were extracted from 18-d-old seedlings of wild-type plants (Col) and *35S*:*RTL1-Flag* (*RTL1-Flag*) plants and hybridized with an anti-RTL1 antibody. Hybridization with an anti-RPL13 antibody serves as a loading control. **F)** Immunostaining of root cells from 8-d-old seedlings of wild-type plants (Col) and *35S*:*RTL1-Flag* (*RTL1-Flag*) plants was performed using an anti-RTL1 antibody and revealed with Alexa 488.

In *Arabidopsis* wild-type plants grown under laboratory conditions, RTL2, RTL4, and RTL5 are expressed throughout the plant. In contrast, RTL1 is weakly expressed in roots, while RTL3 is below detectable levels in all tested tissues ([19,23] and http://jsp.weigelworld.org/expviz/expviz.jsp). Moreover, RTL1 and RTL3 have no known biological or biochemical function. Given that several key components of antiviral RNAi, including the *Arabidopsis* AGOs AGO1, AGO2, and AGO5 and rice AGO2 and AGO18, are induced during virus infection [24–27], and given that the RTL1 and RTL3 enzymes harbor RNaseIII and DRB domains that could bind to and cleave dsRNA intermediates of virus replication, we reasoned that RTL1 or RTL3 could help plants to fight against viruses if their expression is induced during virus infection and if they are expressed where viruses replicate. To test this hypothesis, we inoculated wild-type *Arabidopsis* (Col) plants with different single-stranded RNA viruses: turnip crinkle virus (TCV, a Carmovirus), turnip vein clearing virus (TVCV, a Tobamovirus), cucumber mosaic virus (CMV, a Cucumovirus), and turnip yellow mosaic virus (TYMV, a Tymovirus), and we measured the accumulation of *RTL1* and *RTL3* mRNA. The accumulation of *RTL2* mRNA was also measured to examine a possible contribution of this protein during virus infection because it also harbors RNaseIII and DRB domains and is weakly expressed under regular conditions [19,23]. Three weeks after inoculation, an ~20-fold increase in *RTL1* mRNA accumulation was observed in systemically infected leaves, whereas the level of *RTL2* and *RTL3* mRNA remained largely unchanged (Fig 1A). This ~20-fold increase in *RTL1* mRNA accumulation was observed after individual infection with the four viruses, indicating that increased *RTL1* expression is a general response to diverse virus infections.

Then, we analyzed the subcellular localization of RTL1 to determine if it accumulates in the cytoplasm where RNA viruses replicate. To this end, a *35S*:*RTL1-GFP* construct was transiently introduced into onion epidermal cells by biolistic methods. GFP imaging revealed the presence of RTL1 in both cytoplasmic and nuclear compartments (Fig 1D). In addition, an antibody directed against RTL1 was produced in rats (see Material and Methods), and the specificity of the antibody was tested by western blot analysis (Fig 1E). No signal was detected in wild-type seedlings, whereas a single band of the expected size was detected in transgenic *Arabidopsis* plants ectopically expressing an epitope-tagged RTL1 protein (*35S*:*RTL1-Flag* plants, see below). Then, RTL1 localization was analyzed by immunostaining of root cells (see Material and Methods). A weak signal was detected in wild-type roots (Fig 1F), consistent with *RTL1* mRNA being weakly expressed in roots of wild-type plants (Fig 1B). A stronger signal was detected in *35S*:*RTL1-Flag* plants (Fig 1F), consistent with RTL1 being more abundant in *35S*:*RTL1-Flag* plants than in wild-type plants (Fig 1E). Immunostaining imaging revealed that the RTL1 protein is excluded from the nucleolus, but present in the nucleoplasm and in the cytoplasm (Fig 1F).

### Plants Overexpressing RTL1 Lack ta-siRNAs, endoIR-siRNAs, p4-siRNAs, and Certain Young miRNAs

**Fig 2:**
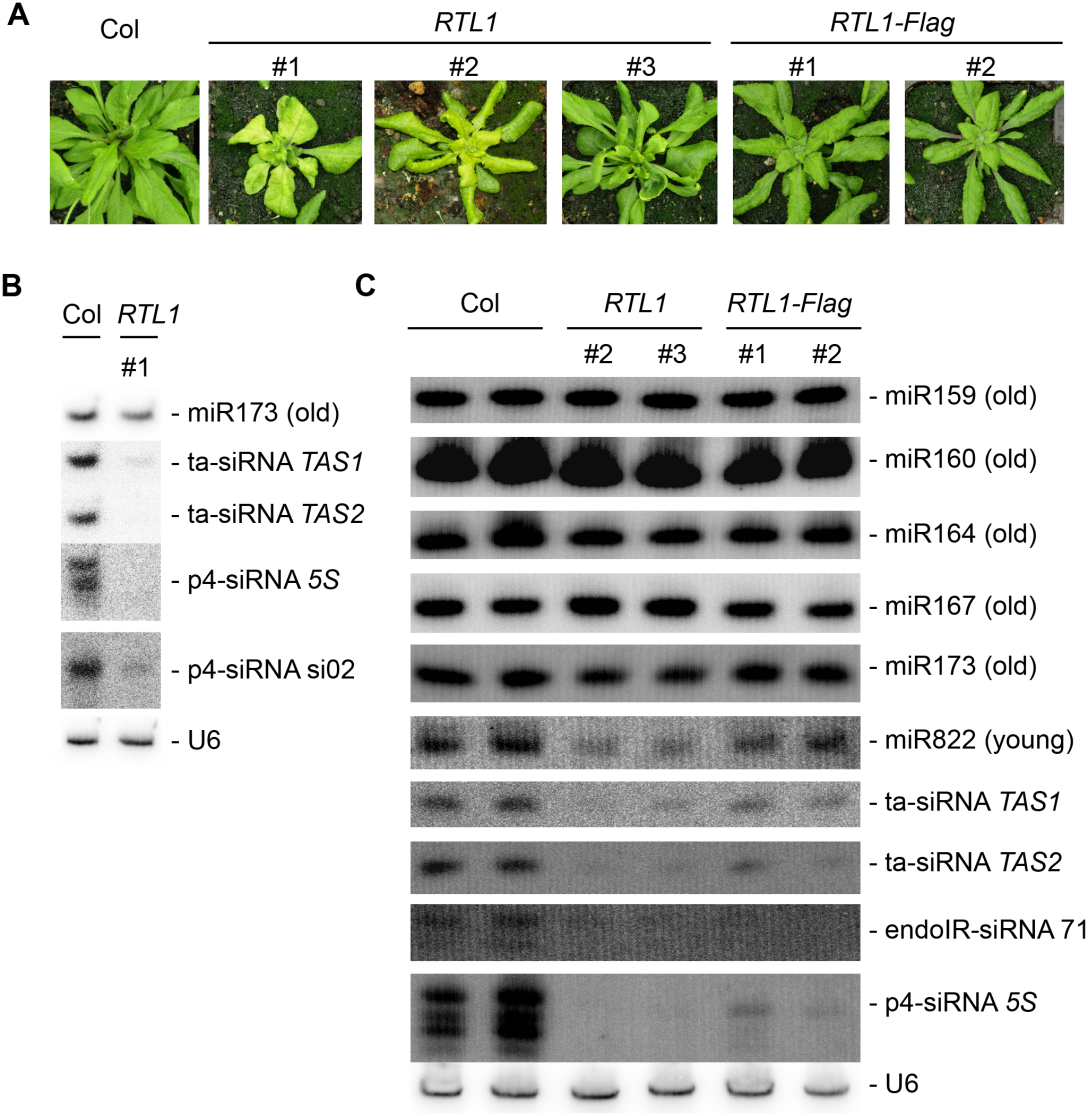
Phenotype of *35S*:*RTL1* plants and RNA gel blot analysis of small RNAs. **A**) Range of phenotypes of transgenic plants overexpressing RTL1. A wild-type plant (Col) is shown as control. **B**) and **C**) Gel blots of total RNA from flowers of wild-type (Col), *35S*:*RTL1* (*RTL1*) and *35S*:*RTL1-Flag* (*RTL1-Flag*) plants were hybridized with the indicated probes. For B, a single blot was successively hybridized, stripped, and rehybridized with the different probes. For C, ten identical blots were hybridized each with a different probe and then rehybridized with U6 as a loading control. A representative U6 control is shown. All U6 controls can be seen in S1 Fig.

Because viruses cause many changes at the developmental and molecular level, we decided to gain specific insight into the biochemical function of RTL1 by analyzing *Arabidopsis* transgenic plants ectopically expressing RTL1 (*35S*:*RTL1*) instead of virus-infected plants. These plants exhibited a range of developmental defects, including smaller stature, yellowing and curled rosette leaves, late flowering, and sterility (Fig 2A). We also produced transgenic plants overexpressing RTL1 proteins tagged with Flag or Myc epitopes and found that they generally exhibited milder or no developmental defects (Fig 2A and S9 Fig), suggesting reduced activity of RTL1-tagged proteins. Progeny plants that had inherited the *35S*:*RTL1* or *35S*:*RTL1-tag* transgene exhibited the same developmental defects as their parents, whereas siblings that had segregated away the transgene looked like wild-type plants, indicating that developmental defects are directly caused by RTL1 overexpression

To determine if the small RNA repertoire of *35S*:*RTL1* plants is modified, RNA gel blot analyses were performed on flowers of three independent *35S*:*RTL1* transformants and on the two *35S*:*RTL1-Flag* transformants that were analyzed by western blot (Fig 1E). Hybridization with representative miRNA and siRNA probes showed that levels of conserved (old) miRNA are largely unchanged in *35S*:*RTL1* plants, whereas the levels of siRNAs are strongly reduced (Fig 2B and 2C). Note that the two *35S*:*RTL1-Flag* plants exhibit a lower decrease in siRNA levels (Fig 2C), which together with their reduced developmental defects suggests reduced stability or activity of the RTL1-Flag fusion protein (see below).

**Fig 3:**
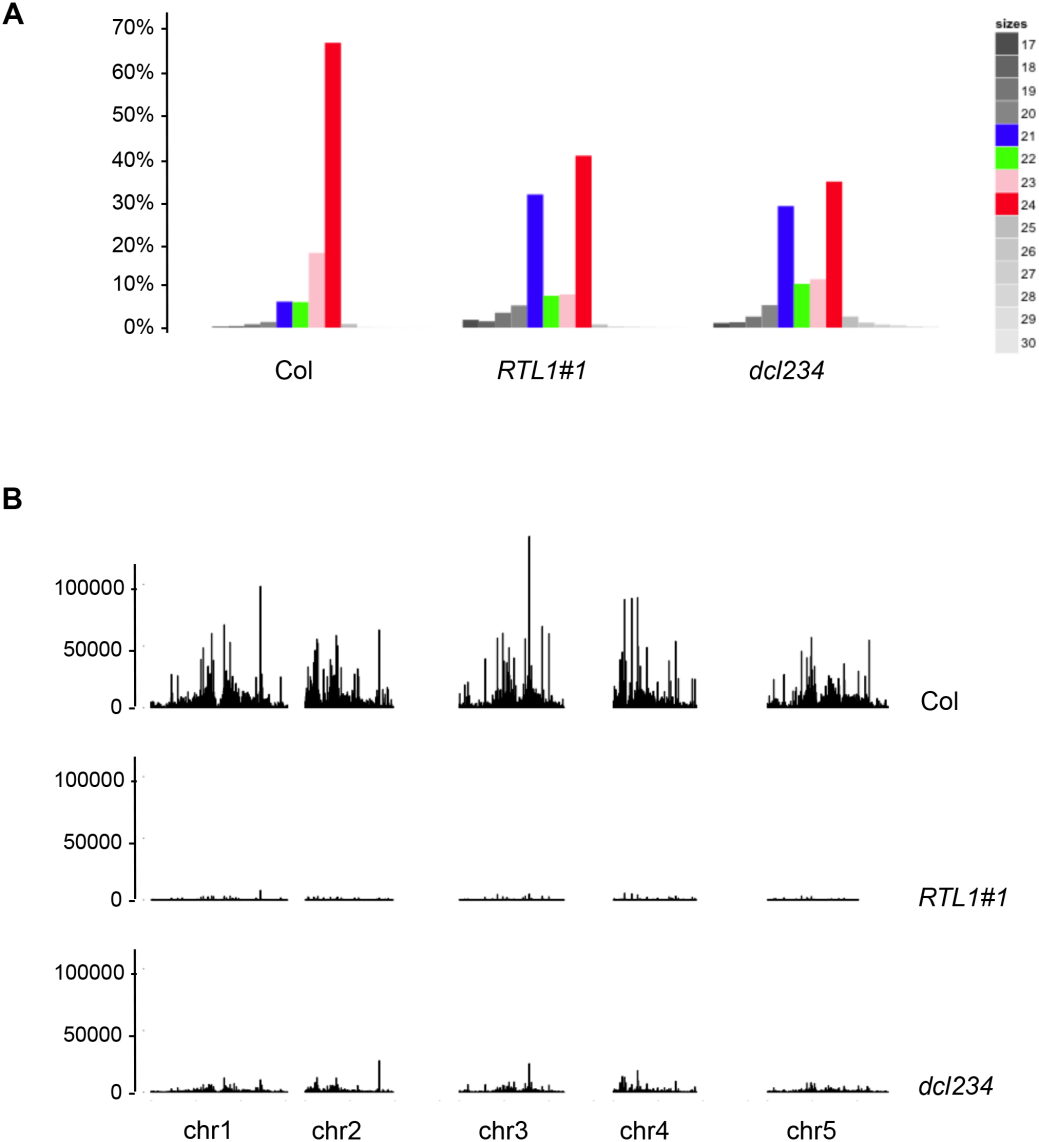
Plants overexpressing RTL1 have reduced global levels of siRNAs. Small RNAs from flowers of wild-type (Col), *dcl2dcl3dcl4 (dcl234)* mutant and the *35S*:*RTL1 (RTL1)* plant #1 analyzed in Fig 2 were subjected to high throughput sequencing. **A**) Size distribution of reads that perfectly match the *Arabidopsis* nuclear genome, excluding rRNA and tRNA. The proportion of each size of small RNA is indicated by a different color: 21 nt (blue), 22 nt (green), 23 nt (pink) and 24 nt (red) and a gradient of grey for 17 to 20 nt and 25 to 30 nt. **B**) Normalized abundance of siRNAs spanning the nuclear genome. Only siRNAs matching a unique genomic location without ambiguity were considered. Normalization was made to the total of conserved miRNAs.

To obtain genome-wide small RNA profiling, small RNAs were sequenced from flowers of the *35S*:*RTL1* line #1. A wild-type Col control and a *dcl2 dcl3 dcl4* triple mutant (*dcl234*) were also sequenced for comparison. Flowers were chosen because it is the tissue producing the highest variety of small RNAs. The distribution of the 17 to 30 nt reads matching the nuclear *Arabidopsis* genome but excluding tRNA and rRNA sequences was examined (S1 Table). Remarkably, the small RNA size distribution in *35S*:*RTL1* plants strongly differed from that in Col, and in fact resembled that in *dcl234* (Fig 3A). Because conserved miRNAs accumulate to similar levels in *35S*:*RTL1* and wild-type plants (Fig 2C), sequence data were normalized to the 27 DCL1-dependent miRNAs that define the 22 conserved miRNA families [28]. Analysis of the normalized chromosomal distribution of unique small RNA reads that do not correspond to conserved miRNAs, i.e., primarily siRNAs, revealed that *35S*:*RTL1* plants almost entirely lack siRNAs (Fig 3B).

**Fig 4:**
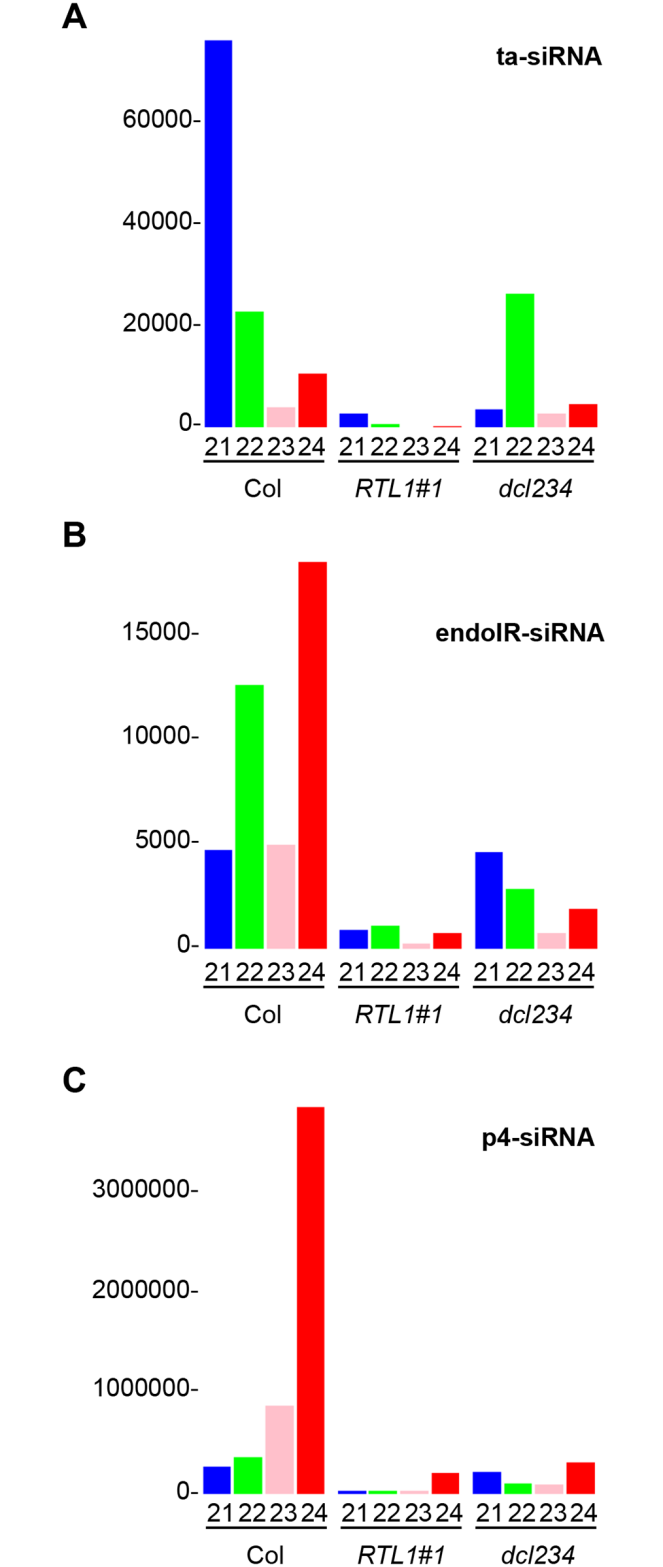
Normalized abundance of the three major classes of endogenous siRNAs. **A**) ta-siRNAs. **B**) endoIR-siRNas. **C**) PolIV/PolV-siRNAs. Small RNAs from wild-type (Col), *35S*:*RTL1* (*RTL1*) transgenic plants and *dcl2dcl3dcl4 (dcl234)* mutants were classified as ta-siRNAs, endoIR-siRNas, or PolIV/PolV-siRNAs based on published annotation. Small RNA abundance was normalized to the total amount of conserved miRNAs. Each size of small RNA is indicated by a different color: 21 nt (blue), 22 nt (green), 23 nt (pink), and 24 nt (red).

Sorting siRNAs into their major functional categories showed that RTL1 overexpression strongly impacts the accumulation of ta-siRNAs, endoIR-siRNAs, and p4-siRNAs (see Fig 4 for whole-genome analysis and S2, S3, and S4 Figs for the analysis of ta-siRNAs, endoIR-siRNAs, and p4-siRNAs representative loci). The level of several young miRNAs was also analyzed because their dsRNA precursors are intermediate between the long precursors of endoIR-siRNAs and the short precursors of canonical miRNAs. RNA gel blot analysis showed reduced accumulation of one young miRNA, miR822, in *35S*:*RTL1* plants (Fig 2C). For other young miRNAs, which are less abundant, sequencing data were analyzed. The levels of mature miR822, miR833, miR838, and miR869 were reduced in *35S*:*RTL1* plants (S5 Fig), suggesting that RTL1 has the same effect on young miRNAs and siRNAs. Nevertheless, *35S*:*RTL1* plants overaccumulated small RNAs from the *MIR828*, *MIR835*, *MIR839*, and *MIR862* loci, and these small RNAs did not always correspond to the mature miR828, miR835, miR839, and miR862 found in Col (S6 Fig), indicating that RTL1 could promote the accumulation of novel small RNA species at discrete loci.

The fact that novel small RNAs are overproduced from *MIR828*, *MIR835*, *MIR839*, and *MIR862* loci in *35S*:*RTL1* plants prompted us to determine how many loci over-accumulate small RNAs in *35S*:*RTL1* plants. For this, the accumulation of small RNAs was analyzed on 100 bp sliding windows. Sliding windows containing at least 100 reads in either Col or *35S*:*RTL1* plants and showing a different accumulation between Col and *35S*:*RTL1* plants (*p* < 0.005) were identified. Assembling overlapping sliding windows defined 10,814 nonoverlapping regions showing a decreased accumulation of small RNAs in *35S*:*RTL1* plants and only 16 regions showing an increased accumulation of small RNAs in *35S*:*RTL1* plants (S2 Table). Further clustering based on genome annotation and proximity of differentially expressed regions (maximal distance admitted between nonoverlapping regions = 1 kb) defined 6,089 and 13 loci showing decreased and increased accumulation of small RNAs in *35S*:*RTL1* plants, respectively (S3 Table). Details about the 13 loci for which small RNA accumulation was increased in *35S*:*RTL1* plants can be found in S1 Text and in supplemental S6, S7, and S8 Figs.

### RTL1 Inhibits Transgene PTGS

**Fig 5:**
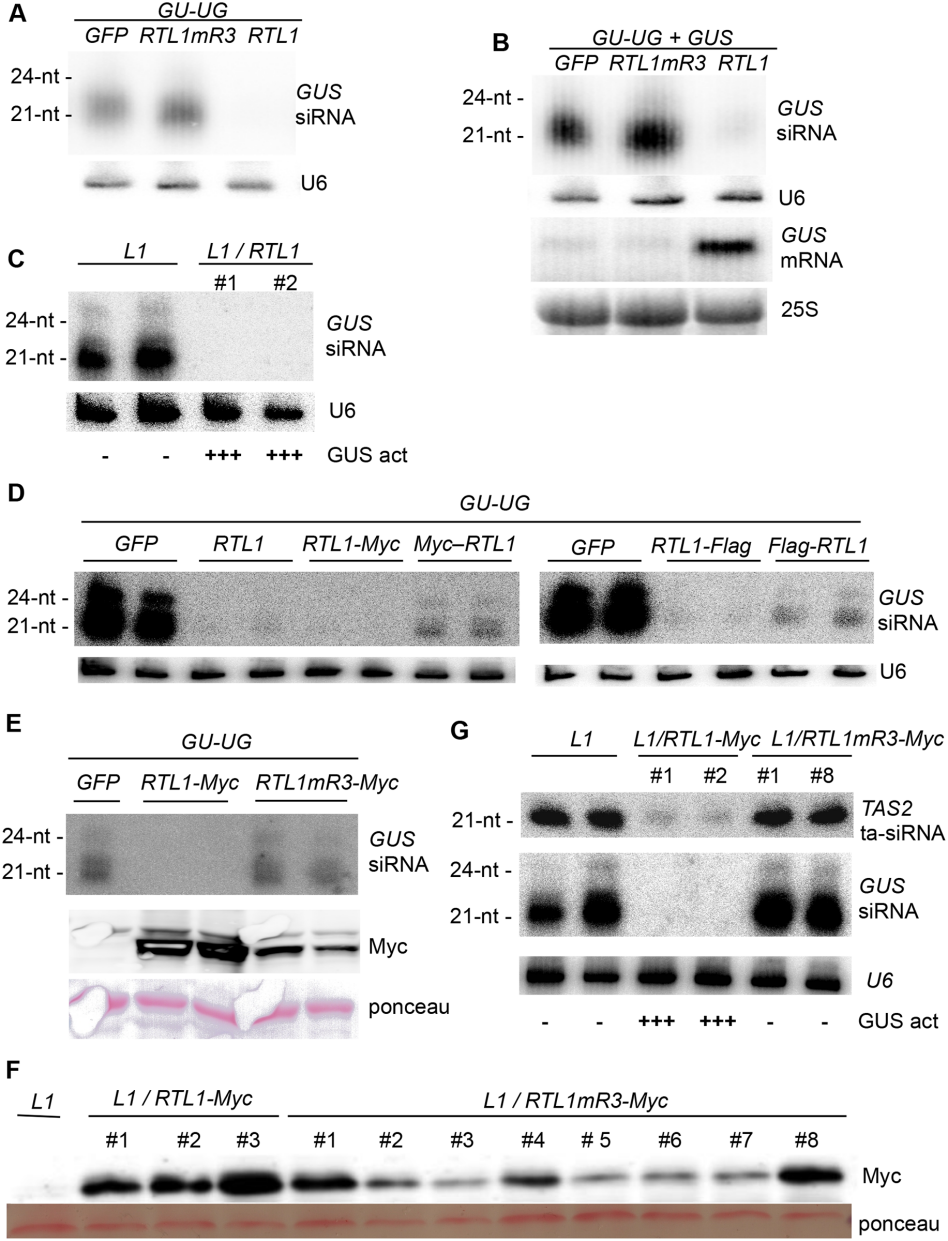
The RTL1 RNaseIII domain is required for inhibition of transgene PTGS. **A**) *N*. *benthamiana* leaves were infiltrated with a *35S*:*GU-UG* construct (*GU-UG*) together with either a wild-type *35S*:*RTL1* construct *(RTL1)*, a construct mutated in the RNaseIII domain (*RTL1mR3*) or a *35S*:*GFP* control *(GFP)*. Low molecular weight (LMW) RNAs were hybridized with a *GUS* probe and with *U6* as a loading control. **B**) *N*. *benthamiana* leaves were infiltrated with a *35S*:*GU-UG* construct (*GU-UG*) and a *35S*:*GUS* construct (*GUS)* together with either a wild-type *35S*:*RTL1* construct *(RTL1)*, a construct mutated in the RNaseIII domain (*RTL1mR3*), or a *35S*:*GFP* control *(GFP)*. LMW RNAs were hybridized with a *GUS* probe and with U6 as loading control. High molecular weight RNAs were hybridized with a *GUS* probe and with 25S as loading control. **C**) The *Arabidopsis* line *L1* carrying a *35S*:*GUS* transgene silenced by PTGS was transformed with a wild-type *35S*:*RTL1* construct *(RTL1)*. LMW *GUS* RNAs from two independent transformants were analyzed. Note that the images presented in this panel are internal to the images presented in S9C Fig. **D**) *N*. *benthamiana* leaves were infiltrated with a *35S*:*GU-UG* construct (*GU-UG*) together with either a wild-type *35S*:*RTL1* construct *(RTL1)*, tagged constructs (*RTL1-Myc*, *Myc-RTL1*, *RTL1-Flag*, *Flag-RTL1*), or a *35S*:*GFP* control *(GFP)*. LMW RNAs were hybridized with a *GUS* probe and with *U6* as a loading control. **E**) *N*. *benthamiana* leaves were infiltrated with a *35S*:*GU-UG* construct (*GU-UG*) together with either a wild-type tagged *35S*:*RTL1-Myc* construct *(RTL1-Myc)*, a construct mutated in the RNaseIII domain (*RTL1mR3-Myc*) or a *35S*:*GFP* control *(GFP)*. LMW RNAs were hybridized with a *GUS* probe and with *U6* as a loading control. Proteins were extracted and hybridized with an anti-Myc antibody. Ponceau staining serves as a loading control. **F**) The *Arabidopsis* line *L1* was transformed with either a wild-type-tagged *35S*:*RTL1* construct *(RTL1-Myc)* or a tagged construct mutated in the RNaseIII domain (*RTL1mR3-Myc*). Proteins were extracted from three independent *RTL1-Myc* transformants and eight independent *RTL1mR3-Myc* transformants and hybridized with an anti-Myc antibody. Ponceau staining serves as a loading control. **G**) LMW RNAs from *RTL1-Myc* and *RTL1mR3-Myc* transformants expressing comparable amount of proteins were hybridized with *GUS* and *TAS2* probes and with *U6* as a loading control.

Given the ability of RTL1 to inhibit the accumulation of endogenous siRNAs at 6,089 out of 6,102 loci, we examined whether RTL1 overexpression also impacted transgene-derived siRNAs. To this end, a *35S*:*GU-UG* transgene expressing an inverted repeat hairpin RNA consisting of the 5’ part of the *GUS* reporter sequence (*GU*) was introduced transiently into *Nicotiana benthamiana* leaves together with either *35S*:*RTL1* or a *35S*:*GFP* control. As expected, *35S*:*GU-UG* + *35S*:*GFP* infiltrated leaves accumulated high levels of 21 and 24 nt *GU* siRNAs, whereas *35S*:*GU-UG* + *35S*:*RTL1*-infiltrated leaves did not accumulate detectable levels of *GU* siRNAs (Fig 5A), indicating that RTL1 prevents the accumulation of transgene siRNAs deriving from an inverted repeat. We also determined the effect of RTL1 on post-transcriptional gene silencing (PTGS), by coinfiltrating the *35S*:*GU-UG* construct and a target *35S*:*GUS* reporter with either *35S*:*RTL1* or a *35S*:*GFP* control. Leaves infiltrated with *35S*:*GU-UG* + *35S*:*GUS* + *35S*:*GFP* lacked *GUS* mRNA, indicating that PTGS of *GUS* was established, whereas *35S*:*GU-UG* + *35S*:*GUS* + *35S*:*RTL1*-infiltrated leaves accumulated *GUS* mRNA (Fig 5B), indicating that RTL1 prevents transgene siRNA-mediated PTGS of the *35S*:*GUS* target. RTL1-mediated inhibition of transgene PTGS was confirmed in the *Arabidopsis* line *L1*, which undergoes spontaneous PTGS of a stably integrated *35S*:*GUS* reporter transgene. Transgenic *L1/35S*:*RTL1* plants exhibited high GUS activity and lacked *GUS* siRNAs, whereas *L1* controls lacked GUS activity and accumulated *GUS* siRNAs (Fig 5C). The high GUS activity observed in *L1/35S*:*RTL1* plants indicates that the release of *L1* PTGS does not result from transcriptional interference between 35S promoters, and thus directly results from the effect of RTL1.

### RTL1 Activity Requires a Functional RNaseIII Domain

To determine if an intact RNaseIII domain is required for RTL1 to inhibit siRNA accumulation and PTGS, amino acids E89 and D96 of RTL1, which are integral to the conserved catalytic site of RNaseIII enzymes and are required for RNaseIII activity [10], were both mutagenized to alanine (resulting in the mutant protein RTL1mR3). Then, *N*. *benthamiana* leaves were coinfiltrated with *35S*:*GU-UG* and either *35S*:*RTL1* or *35S*:*RTL1mR3*. *35S*:*GU-UG* + *35S*:*RTL1mR3*-infiltrated leaves accumulated high levels of 21 and 24 nt *GU* siRNAs, similar to *35S*:*GU-UG* + *35S*:*GFP*-infiltrated leaves, whereas *35S*:*GU-UG + 35S*:*RTL1*-infiltrated leaves lacked *GU* siRNAs (Fig 5A), indicating that the wild-type RNaseIII domain of RTL1 is required for RTL1 to impact siRNA accumulation. Next, to determine the impact on PTGS, *N*. *benthamiana* leaves were coinfiltrated with *35S*:*GU-UG* + *35S*:*GUS* and either *35S*:*RTL1* or *35S*:*RTL1mR3*. *35S*:*GU-UG + 35S*:*GUS* + *35S*:*RTL1mR3*-infiltrated leaves silenced *GUS* and accumulated *GUS* siRNAs and lacked *GUS* mRNA, similar to *35S*:*GU-UG + 35S*:*GUS + 35S*:*GFP*-infiltrated leaves, whereas *35S*:*GU-UG + 35S*:*GUS + 35S*:*RTL1*-infiltrated leaves lacked *GUS* siRNAs and accumulated *GUS* mRNA (Fig 5B), indicating that an intact RTL1 RNAseIII domain is necessary to suppress PTGS.

To monitor RTL1 and RTL1mR3 protein accumulation, two different epitope tags were added individually to RTL1 as C-terminal or N-terminal fusions, and then the RNaseIII domain was mutagenized. To determine if the presence of the epitope tags impaired wild-type RTL1 function, the *35S*:*GU-UG* construct was infiltrated into *N*. *benthamiana* leaves with either *35S*:*RTL1*, *35S*:*Flag-RTL1*, *35S*:*RTL1-Flag*, *35S*:*Myc-RTL1* or *35S*:*RTL1-Myc* constructs. The *35S*:*RTL1-Flag* and *35S*:*RTL1-Myc* constructs reduced *GU* siRNA accumulation almost as efficiently as the *35S*:*RTL1* control, whereas the *35S*:*Flag-RTL1* and *35S*:*Myc-RTL1* constructs had a weaker effect (Fig 5D), suggesting that the addition of these N-terminal tags impacted RTL1 activity. Consistent with this observation, *35S*:*Flag-RTL1* and *35S*:*Myc-RTL1 Arabidopsis* transformants did not exhibit developmental defects, whereas *35S*:*RTL1-Flag* and *35S*:*RTL1-Myc* transformants displayed developmental defects similar to, although less severe than, *35S*:*RTL1* plants (S8A Fig). Indeed, similar to *35S*:*RTL1* plants, both *35S*:*RTL1-Flag* and *35S*:*RTL1-Myc* plants exhibited downward leaf curling, typical of plants lacking *TAS3* ta-siRNAs. However, they grew bigger and greener than *35S*:*RTL1* plants (Fig 2A and S8A Fig) and were more fertile, producing up to 30% of the amount of seeds of a wild-type Col control, whereas the amount of seeds produced by *35S*:*RTL1* plants was generally less than 1% of that produced by Col. The *35S*:*RTL1-Flag* and *35S*:*RTL1-Myc* constructs were retained for further analysis because, among the four tagged constructs tested, they showed the strongest reduction in siRNA accumulation (S8B Fig), and the strongest effect on *L1* PTGS (S7C Fig). Note that the accumulation of *GUS* siRNA in *L1/35S*:*Flag-RTL1* plants confirms that *L1* is not subjected to transcriptional silencing when transformed by 35S-driven transgenes.

To ensure that the absence of developmental and molecular defects in *35S*:*RTL1mR3* plants was not due to destabilization or impaired production of the RTL1 protein, the RNaseIII domain of the *35S*:*RTL1-Myc* construct was mutagenized as previously described, and *N*. *benthamiana* leaves were coinfiltrated with the *35S*:*GU-UG* and either *35S*:*GFP*, *35S*:*RTL1-Myc*, or *35S*:*RTL1mR3-Myc* constructs. *35S*:*GU-UG* + *35S*:*RTL1mR3-Myc*-infiltrated leaves accumulated high levels of 21 and 24 nt *GU* siRNAs, whereas *35S*:*GU-UG + 35S*:*RTL1-Myc*-infiltrated leaves lacked *GU* siRNAs (Fig 5E), similar to the results obtained with nontagged *RTL1* and *RTL1mR3* constructs (Fig 5A). However, western blot analysis revealed that the RTL1mR3-Myc protein accumulated to a lower level than the RTL1-Myc protein in infiltrated leaves (Fig 5E), raising concerns about the effect of the two introduced mutations on protein stability. To address this question, *Arabidopsis L1* plants were transformed with either *35S*:*RTL1-Myc*, or *35S*:*RTL1mR3-Myc*, and the amount of RTL1-Myc and RTL1mR3-Myc protein was determined by western blot in a series of individual transformed plants (Fig 5F). Lines *L1/35S*:*RTL1-Myc* #1 and #2 accumulated levels of RTL1 protein that are comparable with those of lines *L1/35S*:*RTL1mR3-Myc* #1 and #8. Thus, these four lines were further analyzed for endogenous and transgene siRNAs (Fig 5G). Both *L1/35S*:*RTL1-Myc* plants exhibited high GUS activity and lacked *GUS* siRNAs and endogenous ta-siRNAs, whereas *L1/35S*:*RTL1mR3-Myc* plants lacked GUS activity and accumulated *GUS* siRNAs and endogenous ta-siRNAs (Fig 5G). Together, these results indicate that RTL1 requires a functional RNaseIII domain to suppress small RNA accumulation.

### RTL1 Cleaves Near-Perfectly Paired Long dsRNA

The effect of RTL1 on transgene siRNAs, ta-siRNAs, endoIR-siRNAs, p4-siRNAs, and certain young miRNAs could be explained by several, nonexclusive mechanisms:

1. inhibition of the enzymes involved in the biogenesis of their dsRNA precursors,
2. cleavage/degradation of their dsRNA precursors,
3. inhibition of or competition with the DCLs processing their dsRNA precursors
4. cleavage or degradation of these small RNAs.

**Fig 6:**
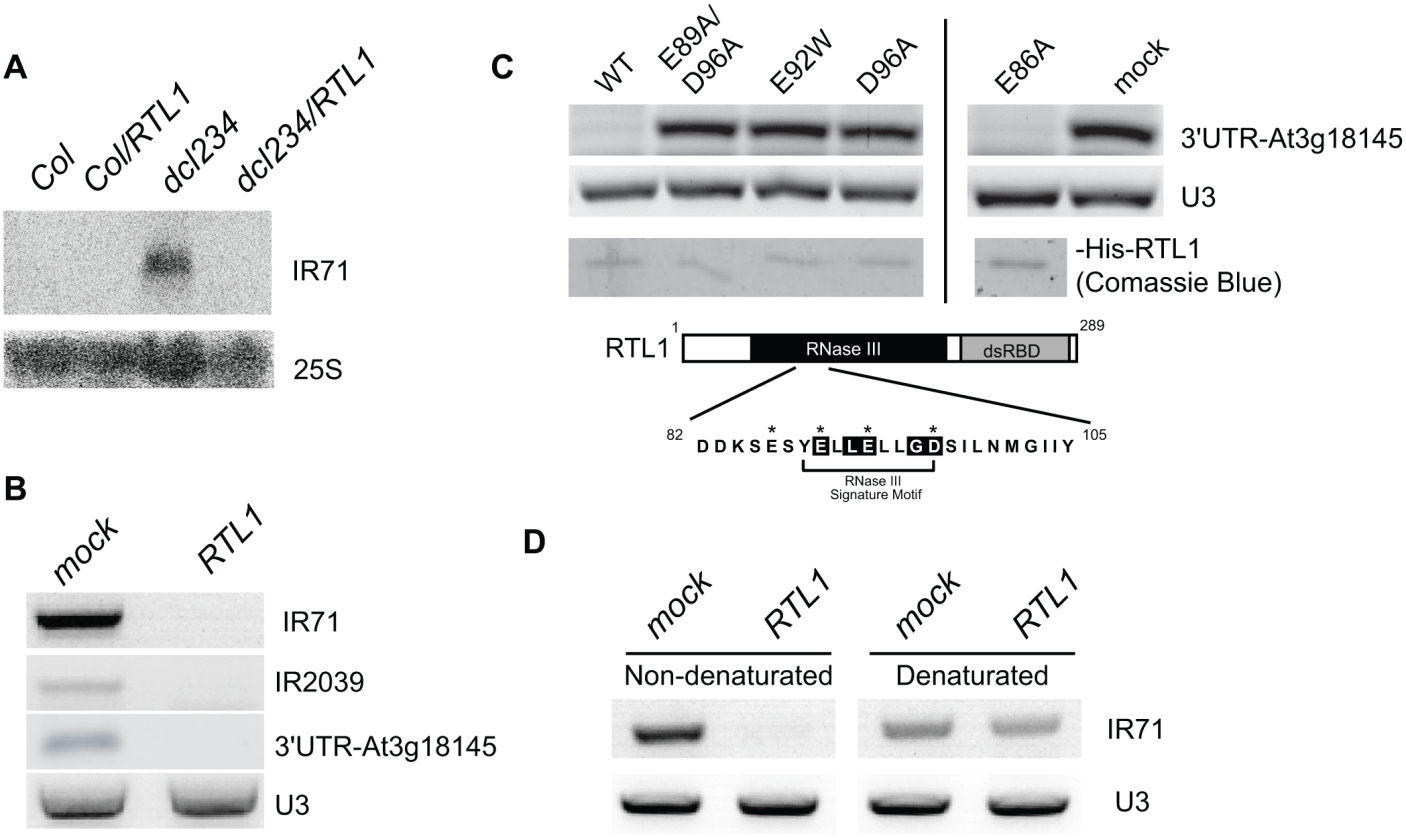
RTL1 cleaves dsRNA. **A**) RNA gel blot detection of *IR71* precursor RNA in wild-type (Col), Col transformed with the *35S*:*RTL1* construct (*Col/RTL1*), *dcl234*, and *dcl234* transformed with the *35S*:*RTL1* construct (*dcl234/RTL1*). Transformants exhibiting a strong RTL1 developmental phenotype were analyzed. High molecular weight (HMW) RNAs extracted from flowers were hybridized with a probe complementary to the *IR71* RNA and with *25S* as loading control. **B**) RNAs extracted from wild-type seedlings were incubated or not with wild-type His-RTL1 and subjected to RT-PCR to detect IR71, IR2039, and At3g18145 (3’UTR) precursor RNAs. **C**) RNAs extracted from wild-type seedlings were incubated with wild-type or mutant His-RTL1 and subjected to RT-PCR reactions to detect At3g18145 (3’UTR) precursor RNAs. Comassie blue-stained gel shows approximately 200 ng of wild-type and mutant proteins. A schematic representation of RTL1 (residues 1–289) is shown at the top. Black and grey boxes correspond to RNaseIII and dsRBD (double stranded RNA Binding Domain) motifs, respectively. The conserved amino acids in the RNase III signature motif are highlighted in black and the residues E86, E89, E92, and D96 mutated in recombinant proteins indicated by an asterisk. **D**) RNAs extracted from wild-type seedlings were denaturated or not before incubation with His-RTL1 and subjected to RT-PCR reactions to detect IR71 precursor RNAs. RT-PCR amplification of U3 snoRNA sequences shows similar amount of RNA in each reaction.

The fact that RTL1 overexpression impairs the accumulation of endoIR-siRNAs and certain young miRNAs rules out that RTL1 inhibits RDR activity. Indeed, endoIR-siRNAs and young miRNAs are processed from self-complementary single-stranded RNAs that do not require RDR for their production. The fact that levels of ta-siRNAs, endoIR-siRNAs, p4-siRNAs, and certain young miRNAs are more strongly reduced in *35S*:*RTL1* plants than in *dcl234* (Fig 4 and S5 Fig) makes it also unlikely that RTL1 inhibits DCL2, DCL3, and DCL4 activity or that RTL1 selectively degrades small RNAs produced by DCL2, DCL3, and DCL4. Indeed, the residual level of siRNAs observed in *dcl234* likely results from a weak activity of DCL1 on siRNA precursors in the absence of DCL2, DCL3, and DCL4, and the disappearance of these siRNAs in *35S*:*RTL1* plants suggests that RTL1 acts upstream of the DCLs, including DCL1. Therefore, it is very tempting to speculate that RTL1 cleaves the dsRNA precursors of ta-siRNAs, endoIR-siRNAs, p4-siRNAs, certain young miRNAs, and transgene siRNAs, thereby preventing their processing by any of the four DCLs. To test this hypothesis, we examined the accumulation of the endoIR-siRNA precursor *IR71* because *IR71*-derived siRNAs are absent in plants expressing the *35S*:*RTL1* construct (Fig 2 and S3 Fig). RNA gel blot analysis revealed that the *IR71* precursor accumulates in flowers of *dcl234* but is below detectable level in wild-type Col, likely because it is efficiently processed by DCL2, DCL3, and DCL4 (Fig 6A). The *IR71* precursor did not accumulate in *dcl234* transformed with the *35S*:*RTL1* construct (Fig 6A), suggesting that it is degraded or destabilized by RTL1.

Confirmation that RTL1 acts on dsRNA was further obtained using an in vitro cleavage assay. A soluble His-RTL1 fusion protein was expressed in *Escherichia coli*, purified to near homogeneity and incubated with total RNA extracted from wild-type *Arabidopsis* seedlings. RT-PCR analysis reveals that purified His-RTL1 cleaves *IR71* and *IR2039* precursor RNAs, which can be visualized by the absence of amplification in the presence of His-RTL1, whereas amplification is detected in RNA fractions incubated without His-RTL1 (Fig 6B). Note that the IR71 precursor can be detected in Col extracts because RT-PCR is more sensitive than the RNA gel blot method used in Fig 6A and/or because DCL2, DCL3, and DCL4 are less expressed in seedlings than in flowers. Purified His-RTL1 also cleaves the hairpin located in the 3’ UTR of At3g18145 from which derives the 24 nt species that replace miR3440 in *35S*:*RTL1* plants (S7 Fig and Fig 6B). The proof that cleavage is due to RTL1 activity and not to bacterial RNaseIII contamination was obtained using His-RTL1 proteins mutated in the RNaseIII domain. For this purpose, His-RTL1 proteins mutated at single (E86A, E92W, D96A) or double (E89A/D96A) positions were used in the in vitro cleavage assay. RT-PCR analysis revealed that His-RTL1 and His-RTL1mR3 (E86A) are able to cleave At3g18145 3’UTR sequences (Fig 6B and 6C). In contrast, amplification was detected in RNA fractions incubated without proteins or incubated with His-RTL1 proteins mutated at residues E92 and D96 (Fig 6C), indicating that RTL1 RNA cleavage activity requires a functional RNase III domain. Finally, the specificity of RTL1 towards dsRNA was demonstrated by incubating His-RTL1 with denatured or non-denatured RNA. Indeed, His-RTL1 was able to cleave non-denatured but not denatured *IR71* precursor RNA (Fig 6D). Together, these results demonstrate that RTL1 cleaves dsRNA. Nevertheless, His-RTL1 activity appears specific to certain dsRNA. Indeed, the abundant and highly structured *U3* snoRNA [29], which is not a siRNA precursor, was not cleaved by His-RTL1 (Fig 6B and 6D), indicating that RTL1 cleavage activity is sequence- and/or structure-specific, or depends on a cofactor that recognizes certain dsRNA such as siRNA precursors.

### VSRs Counteract RTL1 Activity

**Fig 7:**
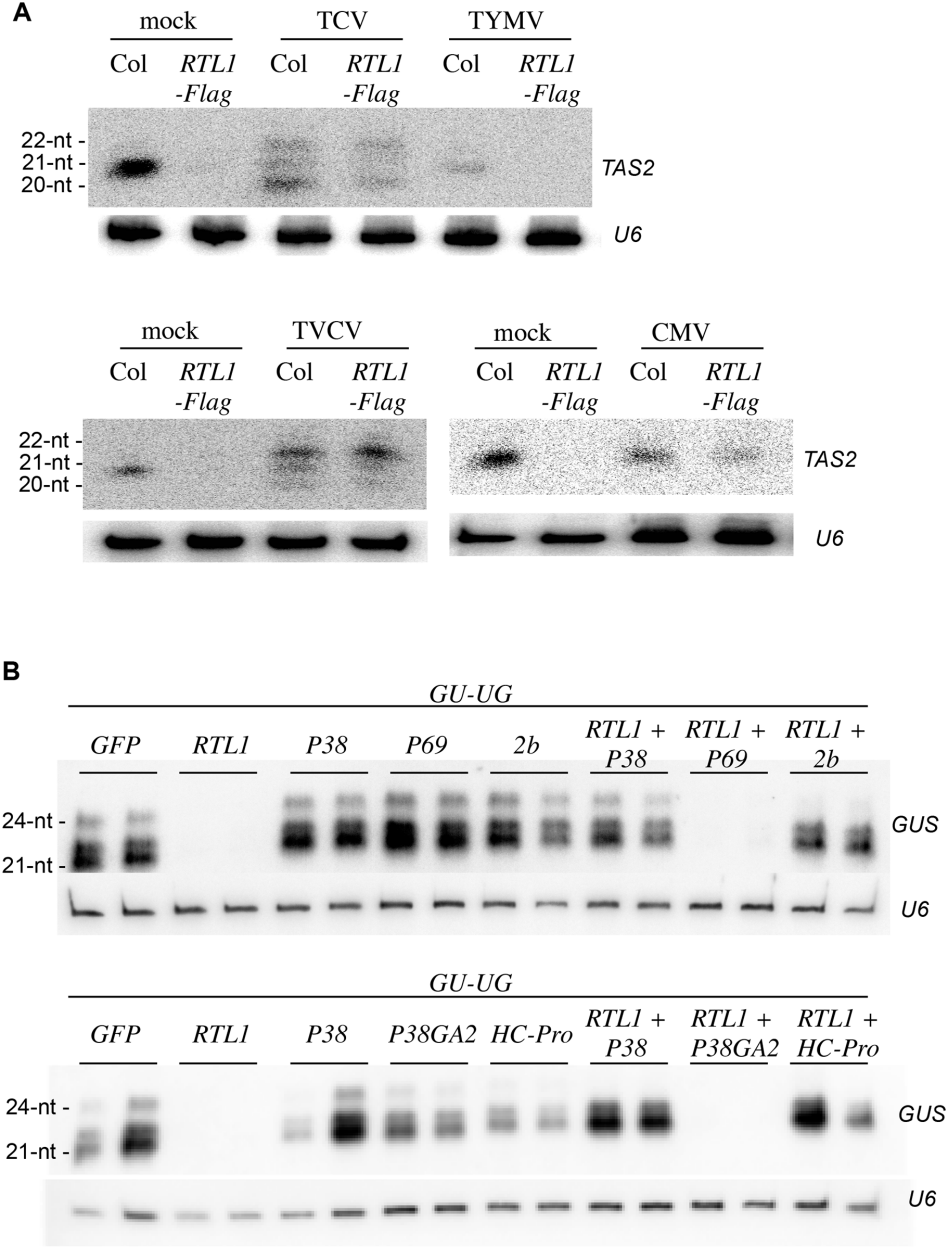
RTL1 activity is suppressed by VSRs. **A**) RNAs extracted from water (mock)- or virus inoculated wild-type (Col) and *35S*:*RTL1-Flag* (*RTL1-Flag*) plant #2 were hybridized with a *TAS2* probe and *U6* as a loading control. **B**) *N*. *benthamiana* leaves were infiltrated with a *35S*:*GU-UG* construct (*GU-UG*) together with either a *35S*:*GFP* control *(GFP)*, a wild-type *35S*:*RTL1* construct *(RTL1)*, a *35S*:*VSR* construct (*P38*, *P38GA2*, *P1/HC-Pro*, *2b*, *or P69*), or a mix of the *35S*:*RTL1* and *35S*:*VSR* constructs. LMW RNAs were hybridized with a *GUS* probe and with *U6* as a loading control.

Upon virus inoculation, *RTL1* mRNA overaccumulates in systemic leaves (Fig 1A). To determine if this level of RTL1 induction impacts siRNA production, the accumulation of endogenous *TAS2* ta-siRNAs was compared in water (mock)-, TCV-, TVCV-, CMV-, and TYMV-inoculated Col plants. *TAS2* ta-siRNA accumulation was strongly reduced in TYMV-inoculated plants (Fig 7A). This reduction was similar to that observed in *35S*:*RTL1-Flag* plants (Fig 2C), indicating that the RTL1 level in TYMV-infected plants impacts siRNA production. In contrast, *TAS2* ta-siRNAs accumulated in TCV-, TVCV-, and CMV-inoculated plants almost at Col levels (Fig 7A). Note that the heterogeneity of ta-siRNA size observed in TCV- and TVCV-inoculated plants is consistent with previous reports [30–32]. These results suggest that despite a similar induction of *RTL1* mRNA accumulation during infection by all four viruses, TCV, TVCV, and CMV, but not TYMV, seem to prevent the effect of RTL1 on endogenous siRNA accumulation. To test if TCV, TVCV, and CMV actually block RTL1 activity, the effect of these viruses on the accumulation of endogenous *TAS2* ta-siRNAs was examined in *35S*:*RTL1-Flag#2* plants because these plants constitutively overaccumulate RTL1 protein (Fig 1E) and exhibit reduced siRNA accumulation (Fig 2C). *TAS2* ta-siRNAs accumulated in TCV-, TVCV-, and CMV-inoculated *35S*:*RTL1-Flag#2* plants at a level near that of TCV-, TVCV-, and CMV-inoculated Col plants (Fig 7A), indicating that TCV, TVCV, and CMV block the activity of either virus-induced wild-type RTL1 protein or ectopically expressed RTL1-Flag protein. *TAS2* ta-siRNA accumulation was similarly abolished in mock- and TYMV-inoculated *35S*:*RTL1-Flag#2* plants (Fig 7A), indicating that TYMV does not block RTL1 activity.

The results above indicate that *RTL1* expression is induced in response to virus infection, but that most viruses have evolved strategies to inhibit RTL1 activity, suggesting that RTL1 could be deleterious for virus replication. It has long been known that most viruses encode proteins that inhibit components of the plant antiviral PTGS defense. These proteins are referred to as VSRs. Until now, inhibition of plant PTGS by VSR proteins was considered sufficient to successfully establish infection. However, our results suggest that viruses also inhibit RTL1 activity, suggesting that inhibition of both RTL1 and PTGS is necessary to successfully establish infection. This hypothesis raised the question of how viruses inhibit RTL1 activity. To address this question, we first tested if VSR proteins were implicated in the inhibition of RTL1 activity. To this end, *N*. *benthamiana* leaves were coinfiltrated with the *35S*:*GU-UG* construct and either *35S*:*GFP*, *35S*:*RTL1*, *35S*:*VSR*, or *35S*:*RTL1 + 35S*:*VSR* constructs. Among the four viruses tested, two, TCV and CMV, encode strong VSRs, P38 and 2b, respectively, whereas TYMV encodes a weak VSR, P69. Given the effect of TVCV on PTGS [33,34], it likely encodes a VSR as strong as P38; however, the TVCV protein that acts as a VSR has not been identified yet and thus could not be tested here. Whereas *35S*:*RTL1*-infiltrated leaves exhibited strongly reduced levels of *GU* siRNAs compared with *35S*:*GFP*-infiltrated leaves, *35S*:*RTL1 + 35S*:*P38* and *35S*:*RTL1 + 35S*:*2b*-infiltrated leaves accumulated *GU* siRNAs at a level similar to that of *35S*:*P38* and *35S*:*2b*-infiltrated leaves (Fig 7B). Moreover, leaves infiltrated with *35S*:*RTL1 + 35S*:*P38GA2*, which expresses a defective form of P38 [35], showed reduced levels of *GU* siRNAs similar to *35S*:*RTL1 + 35S*:*GFP* infiltrated leaves (Fig 7B). These results indicate that active forms of P38 and 2b are able to prevent RTL1-mediated inhibition of transgene-derived siRNAs. In contrast, *35S*:*RTL1 + 35S*:*P69* infiltrated leaves exhibited strongly reduced levels of *GU* siRNAs, similar to *35S*:*RTL1 + 35S*:*GFP* infiltrated leaves (Fig 7B), indicating that P69 has no effect on RTL1 activity. To extend our analysis of the effects of VSR proteins, we also tested the effect of the strong VSR P1/HC-Pro encoded by turnip mosaic virus (TuMV, a Potyvirus), knowing that TuMV suppresses PTGS as efficiently as TCV and TVCV [33,34]. *35S*:*RTL1 + 35S*:*P1/HC-Pro*-infiltrated leaves accumulated *GU* siRNAs at a level similar to that of *35S*:*P1/HC-Pro*-infiltrated leaves (Fig 7B), indicating that P1/HC-Pro is as efficient as P38 and 2b in suppressing RTL1 activity. Together, these results suggest that every strong VSR is capable of suppressing RTL1 activity.

### RTL1 Overexpression Caused a Severe Aggravation of TYMV-Induced Symptoms

To further examine the biological role of RTL1 in plant-virus interactions, we attempted to compare virus-sensitivity in Col and *rtl1* mutants. However, *rtl1* mutants could not be found in publicly available libraries. Moreover, neither of two artificial miRNAs directed against RTL1 (amiR-RTL1a and amiR-RTL1b) were able to suppress the RTL1 overexpression phenotype conferred by the *35S*:*RTL1* construct, despite an abundant accumulation of the two amiRNAs (S10 Fig). We also attempted to silence *RTL1* by crossing overexpressing lines with lines expressing antisense RNA, but no suppression of the overexpression phenotype was observed, suggesting that *RTL1* mRNA is reluctant to silencing. At last, a CRISPR strategy was used to obtain an *rtl1* mutant, but no deletion within *RTL1* was observed among 68 transformants analyzed, raising the question of whether *rtl1* mutants are viable or produce viable gametes. Therefore, we attempted to address the biological role of RTL1 in plant–virus interactions by comparing the responses of Col and *35S*:*RTL1-Flag*#2 plants after infection with the four representative viruses used in our previous experiments.

**Fig 8:**
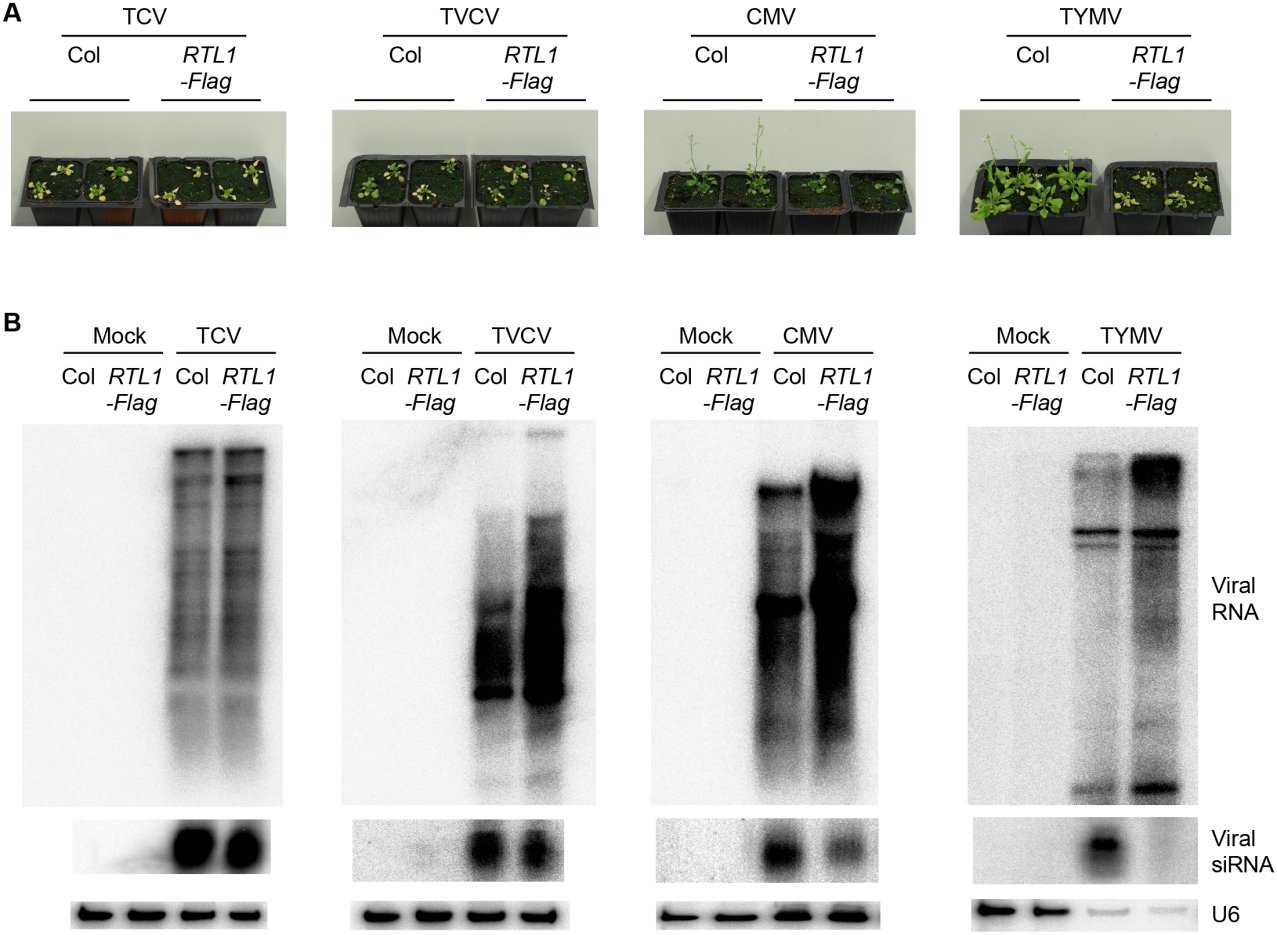
Impact of RTL1 on virus infection. **A**) Pictures of water (mock)-, TCV-, TVCV-, CMV-, and TYMV-inoculated wild-type (Col) and *35S*:*RTL1-Flag* (*RTL1-Flag*) plant #2. Ten-day-old plants were inoculated. Pictures were taken three weeks following inoculation. **B**) RNA gel blot detection of TCV, TVCV, CMV, and TYMV RNAs and siRNAs in total aerial parts of mock- and virus-inoculated wild-type (Col) and *35S*:*RTL1-Flag* (*RTL1-Flag*) plant #2. *U6* was used as a loading control. The *U6* panels for CMV and TVCV are similar to those in Fig 7A because the blots used in Fig 7A were stripped and rehybridized with CMV and TVCV probes to produce Fig 8B.

TCV- and TVCV-inoculated *35S*:*RTL1-Flag* plants developed severe symptoms similar to TCV- and TVCV-inoculated Col (Fig 8A), and all these plants eventually died from the infection. CMV-inoculated *35S*:*RTL1-Flag* developed symptoms milder than TCV- and TVCV-inoculated *35S*:*RTL1-Flag* plants but slightly stronger than CMV-inoculated Col (note that the late flowering phenotype observed in CMV-inoculated *35S*:*RTL1-Flag* plants is due to the late flowering phenotype of *35S*:*RTL1-Flag* plants). Consistent with the symptoms, viral RNA accumulated at high levels in inoculated Col and *35S*:*RTL1-Flag* plants (Fig 8B). Moreover, TCV-, TVCV-, and CMV-derived siRNAs accumulated in both Col- and *35S*:*RTL1-Flag*-inoculated plants (Fig 8B), consistent with the accumulation of endogenous *TAS2* ta-siRNAs in TCV-, TVCV-, and CMV-inoculated *35S*:*RTL1-Flag* plants (Fig 7A), thus confirming that these viruses inhibit RTL1 activity.

In contrast, *RTL1* overexpression caused a severe aggravation of TYMV-induced symptoms (Fig 8A). Indeed, whereas TYMV-inoculated Col only showed mild symptoms and were able to bolt, flower and set seeds, TYMV-inoculated *35S*:*RTL1-Flag* resembled TCV- and TVCV- inoculated Col, which are unable to bolt and which eventually die from the infection (Fig 8A). TYMV-inoculated *35S*:*RTL1-Myc* plants resembled TYMV-inoculated *35S*:*RTL1-Flag* plants, whereas TYMV-inoculated *35S*:*RTL1mR3-Myc* plants behaved like TYMV-inoculated Col (S11 Fig), indicating that the RNaseIII domain of RTL1 is responsible of the aggravated phenotype of TYMV-infected *35S*:*RTL1* plants. Consistent with the aggravation of symptoms, viral RNA accumulated at higher levels in TYMV-inoculated *35S*:*RTL1-Flag* plants compared with TYMV-inoculated Col (Fig 8B). Inversely, TYMV-derived siRNAs did not accumulate in TYMV-inoculated *35S*:*RTL1-Flag*, whereas they accumulate at high level in TYMV-inoculated Col (Fig 8B). These results indicate that, when artificially over-expressed, RTL1 prevents the accumulation of TYMV siRNAs as efficiently as it prevents the accumulation of transgene siRNAs (Fig 5), confirming that TYMV is unable to inhibit RTL1 activity (Fig 7A). Therefore, during TYMV infection, RTL1 acts as an antagonist of the plant antiviral PTGS defense, thus promoting virus proliferation and aggravated symptoms in TYMV-inoculated *35S*:*RTL1-Flag* plants.

## Discussion

Unlike the DICER-type RNAseIII enzymes that produce small RNAs necessary for silencing in a range of eukaryotes, the plant non-DICER RNaseIII enzyme RTL1 does not promote small RNA production but rather represses it. Genome-wide profiling of small RNAs in plants ectopically expressing RTL1 under the control of a strong promoter (*35S*:*RTL1*) revealed that RTL1 inhibits the accumulation of all types of small RNAs normally produced by DCL2, DCL3, or DCL4 from ~6,000 loci. Because RTL1 overexpression does not affect the accumulation of DCL1-dependent conserved miRNAs, which are processed from imperfectly-paired short hairpins, it is likely that RTL1 specifically affects perfectly paired long dsRNA, such as the precursors of ta-siRNAs and p4-siRNAs, which derive from RDR activity, or near-perfectly paired long dsRNA, such as the precursors of young miRNAs and of endoIR-siRNAs. Consistently, in vivo and in vitro assays show that RTL1 cleaves dsRNA. Because siRNAs are almost totally absent in *35S*:*RTL1* plants where the four DCLs are present, RTL1 likely has a very high affinity for long dsRNA and thus cleaves dsRNA before they enter processing by the DCLs. RTL1 constitutive expression does not result in the accumulation of a novel size class of small RNA, indicating that RTL1 does not act as a DCL enzyme and does not produce small RNA per se. Rather, it likely cleaves long dsRNA precursors into multiple fragments, which subsequently are degraded into nucleotides by 5’-to-3’ and/or 3’-to-5’ exoribonucleases. Exceptionally, RTL1-mediated cleavage of dsRNA precursors can give rise to molecules that are better processed by another DCL than the original dsRNA, likely because these cleavage products are stable and/or adopt a conformation that makes them a better substrate for one DCL. For example, the hairpin located at the 3’UTR of *At3g18145* appears to be a bad substrate for DCL1, resulting in a very low production of the 21 nt miR3440b. In contrast, an abundant production of a 24 nt siRNA species is observed in *35S*:*RTL1* plants (S7 Fig), likely because RTL1-mediated cleavage of *At3g18145* mRNA makes it accessible to DCL3. However, the stabilization of a dsRNA following RTL1-mediated cleavage remains exceptional. Indeed, in *35S*:*RTL1* plants, small RNA accumulation is reduced at 6,089 loci whereas it is increased at only 13 loci.

Under laboratory growth conditions, *RTL1* mRNA accumulates at very low levels ([19] and Fig 1). This result explains why siRNAs accumulate in most tissues of wild-type plants, but raises questions about the biological role of RTL1. The fact that RTL1 is conserved in plants (S12 Fig) suggests that it has important functions. Indeed, *rtl1* mutant or *RTL1*-silenced plants have not been identified so far, suggesting that *rtl1* mutants could be impaired in both male and female gamete formation, like *rtl4* mutants [20]. When ectopically expressed, not only does RTL1 prevent the production of endogenous siRNAs, but it also prevents the production of exogenous siRNAs, indicating that RTL1 generally targets long dsRNA. As such, RTL1 could target dsRNA intermediates of viral replication providing that *RTL1* mRNA is induced during virus infection and RTL1 protein expressed where viruses replicate. We indeed observed that RTL1, but not RTL2 or RTL3, is induced in leaves in response to infection by viruses from four different families, consistent with a possible role of RTL1 in plant–virus interactions. Moreover, consistent with an effect of RTL1 on all types of siRNAs, subcellular localization experiments indicated that RTL1 resides in both the cytoplasm and the nucleus, thus allowing a possible interaction of RTL1 with RNA viruses that replicate in the cytoplasm. By cleaving dsRNA intermediates of viral replication, RTL1 would therefore act as a second layer of plant antiviral defense in addition to PTGS. PTGS was primarily deciphered in plants that carried silenced transgenes, but it rapidly became obvious that it is an innate mechanism of antiviral defense in plants, flies, and worms [34,36–38]. Indeed, a fraction of viral RNA is transformed into siRNAs by Dicer enzymes (DCL2 and DCL4 in plants), which, upon loading onto AGO proteins (AGO1 and AGO2 in plants), target viral RNA for destruction [15]. However, this mechanism is rarely efficient to protect plants against infection because most viruses have evolved proteins that suppress PTGS at various steps [17,18,36]. These proteins are referred to as VSRs. VSR proteins often exhibit multiple activities directed against different components, substrates, and/or products of the PTGS machinery, making complex the elucidation of their exact effect on PTGS. For example, TCV P38 binds to AGO1, AGO2, and dsRNA, while CMV 2b binds to AGO1 and dsRNA (for review [17] and references therein). Moreover, VSR proteins do not all localize to the same subcellular compartments. As a consequence, VSR proteins inhibit PTGS at different levels and with different efficiencies. For example, TYMV P69 has limited effect on PTGS [39], whereas CMV 2b, TCV P38, and TuMV P1/HC-Pro efficiently suppress PTGS [33,34]. The VSR encoded by TVCV has not been identified yet, but TVCV suppresses PTGS as efficiently as CMV, TCV, and TuMV [33,34], suggesting that the TVCV VSR is as strong as the CMV, TCV, and TuMV VSRs. Accordingly, TYMV-infected *Arabidopsis* plants exhibit mild symptoms, whereas CMV, TCV, TuMV, and TVCV cause severe symptoms on this plant [33,34,39]. These results collectively suggest that the strength of a VSR determines the efficiency of infection by the corresponding virus. Given that VSR proteins affect several endogenous functions in addition to PTGS (for review [17] and references therein), the outcome of infection could also depend on the effect of VSRs on these endogenous functions. Here, we report that the strong VSRs P38, P1/HC-Pro, and 2b inhibit the activity of the previously uncharacterized RNaseIII RTL1. These results support the hypothesis that RTL1 has evolved as an inducible antiviral plant defense, and that viruses have evolved strategies to inhibit or counteract RTL1 activity like they have evolved strategies to inhibit PTGS. Remarkably, inhibition of RTL1 activity and inhibition of plant PTGS defense appear to rely on the same viral proteins. Whether VSR proteins inhibit PTGS and RTL1 by different or similar mechanisms remains to be determined.

Viruses such as TYMV, which encodes a VSR that has very weak activity against PTGS and no visible effect on RTL1 activity, seem to have evolved a different strategy to escape RTL1. In fact, RTL1 appears to help TYMV to achieve a successful infection. Indeed, *35S*:*RTL1* plants exhibit hypersusceptibility to infection by TYMV. This is probably due to the fact that TYMV-infected *35S*:*RTL1* plants lack TYMV-derived siRNAs, as they lack other siRNAs due to the action of RTL1. As such, TYMV-derived siRNAs cannot target TYMV RNAs for destruction by PTGS. Consistently, TYMV RNAs overaccumulate in TYMV-infected *35S*:*RTL1* plants. How do TYMV dsRNA replication intermediates escape RTL1? One possibility is that TYMV replicates in subcellular compartments from which RTL1 is excluded. Supporting this hypothesis, TYMV replicates in virus-induced chloroplastic membrane vesicles [40]. Another possibility is that TYMV dsRNA replication intermediates simply are not substrates for RTL1 due to particular structure and/or sequence. Therefore, all that RTL1 could do is cleave RDR6-derived TYMV dsRNA, thus preventing the production of TYMV siRNAs that could target TYMV RNA for destruction, resulting in aggravated symptoms in plants that express RTL1.

To conclude, our study has revealed a novel plant protein involved in host–virus interactions. RTL1 likely has evolved as an inducible actor of the cellular defenses that help plants to fight against viruses, likely by degrading dsRNA intermediates of virus replication. However, viruses like CMV, TCV, TuMV, and TVCV have evolved strategies to inhibit RTL1 activity and antiviral PTGS at the same time. In other cases, like for TYMV, the virus does not appear to impede RTL1 activity, and, consequently, RTL1 competes with antiviral PTGS to degrade viral-derived dsRNA that would normally be processed to siRNAs. As such, the strong PTGS defense pathway is sabotaged and TYMV is able to replicate to some extent, suggesting the RTL1-mediated cleavage of viral dsRNAs is less effective at destroying viral RNAs than PTGS, likely due to the absence of siRNA amplification. Further experiments will be required to address the precise action of RTL1 towards each virus.

## Materials and Methods

### Plant Material, Transformation, and Virus Inoculation

*Arabidopsis* plants are in the Col-0 ecotype. The mutant *dcl2dcl3dcl4* and the transgenic line *L1* have been previously described [19,41,42].

Plants were grown in standard long-day conditions (16 h of light and 8 h of dark at 20°C). For comparison of RTL1 expression between roots and leaves of Col, and between leaves of Col and *35S-RTL1-Flag*#2 plants, plants of each genotype were grown vertically for 3 wk in vitro on sterile medium under standard long-day conditions. Leaves and roots of around ten plants grown on the same plate were harvested, and experiments were done on three independent replicates. For northern analyses of endogenous small RNAs and RNA sequencing, plants were grown in soil, and RNAs were extracted from flowers.

For virus infection, 10-d-old in vitro plants were inoculated with TVC, TVCV, CMV, and TYMV 1 d after being transferred to soil. Virus inoculations were performed as previously described [30,34]. Plants were grown for 3 wk under standard long-day conditions, after which the total aerial parts of 4 to 12 plants were harvested. *Arabidopsis* was transformed using the floral-dip method.

For *N*. *benthamiana* agroinfiltrations, 10 ml cultures of *Agrobacterium tumefaciens* C58C1 (pMP90) carrying the plasmids of interest were grown overnight at 28°C and then centrifuged at 6,000 rpm. The bacterial pellets were suspended at 10 mM MgCl2, 10 mM MES pH 5.2, 150 μM acetosyringone solution to a final OD_600_ of 1.0. The resuspended bacterial solution was incubated at 20°C for 3 h and then used to infiltrate leaves. Leaves were harvested 3 d after infiltration.

### Plasmid Constructs and Mutagenesis

For production of *35S*:*RTL1* and *35S*:*RTL1-tagged* constructs, *RTL1* (*At4g15417*) genomic sequence starting at the ATG and ending at the stop codon was PCR-amplified and cloned into the *pDONR207* (Gateway Technology-Invitrogen) using *attB1RTL1F* and *attB2RTL1R* Gateway-adapted oligonucleotides (S4 Table). LR reactions were performed to create the *RTL1* N- or C-terminal *Flag* and *Myc* fusions under the control of the *35S* promoter of the *pGWB* Gateway series. Point mutations in the conserved amino acids of the RTL1 RNaseIII domain were done using QuikChange Site-Directed Mutagenesis Kit (Stratagene). The G89 and D96 amino acids were replaced by an A using the *RTL1m2-F* and *RTL1m2-R* oligonucleotides (S4 Table).

For subcellular localization studies, the RTL1 cDNA sequence was amplified by PCR using primers *5rtl1BspH1* and *3rtl1BspH1* (S4 Table). This fragment was cloned into the NcoI site of the plasmid ppK100 [19] to produce the RTL1-GFP fusion protein.

For production of a His-RTL1 recombinant fusion protein, a full-length RTL1 cDNA was amplified by a reverse transcription polymerase reaction (RT-PCR) as described before [16]. The RTL1 cDNA-encoding amino acids 1 to 289 were cloned into the NdeI/XhoI site of the pET16b plasmid (Novagen, Madison, WI) using primers *5rtl1NdeI* and *3rtl1XhoI* (S4 Table S4to produce His-RTL1 recombinant fusion protein. The mutant versions of His-tagged-RTL1 E86A, E92W, D96A and E89A/D96A were obtained using *5e86*, *3e86*, *5e92*, *3e92*, *5d96*, *3d96*, *5e89d96*, and *3e89d96* primers (S4 Table) with the *Quickchange II Site Directed Mutagenesis Kit* (Agilent Technologies) according to the manufacturer.

### RNA Analyses

RNA extraction and hybridization were performed as previously described [43]. A phosphorimager LAS-4000 Fujifilm was used to quantify the hybridization signals. Oligonucleotides used as probes for small RNA detection are listed in S1 Table. For IR71 precursor RNA detection, a DNA probe was generated by PCR using the IR71fwd1 and IR71rev1 primers. For *RTL1* RT-PCR analysis, cDNA was synthesized using 2 μg of total RNA digested with DNaseI (Fermentas). 500 ng of final DNA-free RNA was used for a reverse transcription with oligo-dT (Fermentas). The PCR was performed using Taq DNA polymerase (Thermo Scientific-Fermentas) and 1 μl of RT reaction using *RTL1-6* and *RTL1-7* oligonucleotides (S4 Table). *EF1α* was used as a standard.

For quantitative RT-PCR, total RNA was extracted using RNeasy plant mini kit (Qiagen). 1 μg of total RNA was digested with DNaseI (Fermentas) and converted to cDNA using the Revertaid H minus reverse transcriptase (Thermo Scientific) and oligo dT. *RTL1*, *RTL2*, *RTL3*, and *GAPDH* expression was measured using Ssoadvanced universal SYBR green supermix (Biorad). Gene expression was normalized to *GAPDH* expression. Primer sequences are listed in S4 Table

### Protein Analyses

Plant protein extraction and western blots using Flag antibodies were performed as previously described [43]. For western blots using RTL1 and RPL13 antibodies, 0.1 g of plant material was homogenized and extracted in 500 μl of 50 mM of Tris-HCl pH 8, 150 mM NaCl, 10 mM EDTA, 50 mM NaF, 1% NP40, 0.5% sodium desoxycholate, 0.1% SDS, 1 mM PMSF, 10 mM βME and 1/100 v/v of antiprotease cocktail inhibitor (Sigma). The extracts were cleared by centrifugation at 13,000 X g for 15 min and conserved at −80°C. Then, SDS-PAGE and Western blot were performed as previously described [44]. The membranes were hybridized with a 1:2,000 of α-RTL1 or with a 1:15,000 dilution of α-RPL13. Signals were revealed using Amersham ECL Select kit following the manufacturer specifications. The rat polyclonal antibody against His-RTL1 fusion protein was customer-made by Eurogentec (Seraing-Belgium). Antibodies against RPL13 were previously described in [45].

### In Vitro Assays

The recombinant His-RTL1 proteins were produced following Novagen's instructions. Briefly, lysis was performed in Binding buffer supplemented with DNase (20 ug/ul), MgCl2 (10 mM final concentration), and Protease inhibitor cocktail (Roche). After cell disruption and centrifugation, the soluble fraction was purified using the Ni2+ resin. Following elution, purified His-RTL1 proteins were finally dialyzed against Sample Buffer (20 mM Tris, pH7,5, 100 mM NaCl, 20% glycerol, 1 mM EDTA, 1 mM DTT).

For the cleavage assay, total RNA was extracted from 14-d-old *A*. *thaliana* plantlets using TriZol reagent (GE Healthcare, Littler Chalfont, Buckinghamshire, UK). After treatment with Turbo DNase (Ambion), 500 ng of RNAs were premixed in reaction Buffer (20 mM Tris, pH 7,5, 50 mM MgCl2), then were incubated with 100 ng of recombinant RTL1 or Sample Buffer, in Reaction Buffer for 10 min at 37°C, the reaction was stopped by heating samples at 70°C for 5 min. 50 ng of treated RNAs were used to realize reverse transcription (RT) (Promega) with IR71rt, IR2039rt: 3'UTRrev and U3rt primers, followed by 42 cycles of PCR with IR71fwd2 and IR71rev2, IR2039fwd and IR2039rev, 3'UTRfwd and 3'UTRrev, and U3fwd and U3rt primers, respectively, using the *Gotaq PCR Kit* (Promega).

For denaturation, the RNA premix was boiled at 70°C for 5 min and immediately placed on ice at least 5 min before adding proteins. Recombinant RTL1 or Sample Buffer were added and incubated for 1 h on ice. Finally, the reaction was stopped by heating samples to 70°C for 5 min. RT reactions were performed with IR71rt and U3rt primers, followed by 42 cycles of PCR with IR71fw2 and IR71rev2 and U3fw and U3rt primers, respectively.

### Bioinformatics

The 3' adapters of the reads were removed with S-MART [46] tools, then sequences were mapped using Mosaik (http://code.google.com/p/mosaik-aligner/) and Bowtie [47] to the TAIR10 assembly. Only unique reads with no mismatch to the genome were kept. Graphs were produced with S-MART tools and *ad hoc* scripts, and indicate normalized read counts. Data were normalized with respect to Col-0 under the assumption that the 27 conserved miRNA (miR156, 157, 159, 160, 162, 164, 165, 166, 167, 168, 169, 170, 171, 172, 319, 390, 391, 393, 394, 395, 396, 397, 398, 399, 403, 408, 472) [28] where globally unaffected (Fig 2). MicroRNA annotation was downloaded from RFAM [48]. EndoIR-siRNAs were annotated using the sRNA-producing loci predicted by [49], which did not match RFAM miRNAs. Loci-producing siRNAs in a PolIV-dependent manner where retrieved from [50]. Young miRNAs were defined as DCL1-independent miRNA: we downloaded the sRNAs produced by [51] and discarded all of those miRNAs that contained at least one read from this data set. The data reported in this paper will be deposited in the Gene Expression Omnibus (GEO) database, www.ncbi.nlm.nih.gov/geo (accession no. GSE49866).

Plant protein sequences homologous to *Arabidopsis* RTL1 were retrieved using TblastN. DCL sequences were discarded based on the presence of RNA helicase and PAZ domains upstream of the RNaseIII and DRB domains. In each species, the RTL1 ortholog was identified based on the presence of a single RNaseIII domain and a single DRB domain. Proteins were aligned using MultiAlign.

### Imaging

For the subcellular localization of RTL1-GFP, onion epidermal layers were transfected using the PDS-1000/He biolistic. GFP microscopic images were taken using a Zeiss Axioskop 2 microscope and recorded using a Leica DC 300 FX digital camera (Leica).

For subcellular localization of native RTL1, immunofluorescence was performed using 8 d-old roots as described previously [44]. Treated roots were incubated at 4°C overnight with a rat anti-RTL1 antibody (1:1,000; Eurogentec), then with antirat coupled with Alexa 488 (1:10,000, Life Technologies), for 3 h at room temperature. Slides were then mounted in Vectashield containing DAPI solution. Observation and Imaging was performed using a confocal microscope LSM 700 from Zeiss.

## Supporting Information

S1 Data Excel spreadsheet containing, in separate sheets, the underlying numerical data and statistical analysis for Figs 1A, 1B, 1C, 3A, 3B, 4A, 4B, 4C, S2, S3, S4, S5, S6, S7 and S8. (XLSX)

S1 Fig RNA gel blot analysis of small RNAs in *35S*:*RTL1* plants. Ten identical gel blots of total RNA from flowers of wild-type (Col), *35S*:*RTL1* (*RTL1*) and *35S*:*RTL1-Flag* (*RTL1-Flag*) plants were hybridized each with a different probe and then rehybridized with U6 as a loading control. A simplified figure showing one representative U6 control is shown in Fig 2C.
(TIF)

S2 Fig Normalized abundance and distribution of ta-siRNAs from four *TAS* loci. Small RNA abundance was normalized to the total amount of conserved miRNAs. The distribution of ta-siRNAs along the chromosome is shown on the left, and the size distribution is shown on the right. The sizes are indicated by different colors: 21 nt (blue), 22 nt (green), 23 nt (pink), and 24 nt (red).
(TIF)

S3 Fig Normalized abundance and distribution of endoIR-siRNAs from two *IR* loci. Small RNA abundance was normalized to the total amount of conserved miRNAs. The distribution of endoIR-siRNAs along the chromosome is shown on the left, and the size distribution is shown on the right. The sizes are indicated by different colors: 21 nt (blue), 22 nt (green), 23 nt (pink), and 24 nt (red).
(TIF)

S4 Fig Normalized abundance and distribution of p4-siRNAs from four *P4* loci. Small RNA abundance was normalized to the total amount of conserved miRNAs. The distribution of PolIV/PolV-siRNAs along the chromosome is shown on the left, and the size distribution is shown on the right. The sizes are indicated by different colors: 21 nt (blue), 22 nt (green), 23 nt (pink), and 24 nt (red).
(TIF)

S5 Fig Normalized abundance and distribution of miRNAs from four young *MIR* loci. Small RNA abundance was normalized to the total amount of conserved miRNAs. The distribution of young miRNAs along the chromosome is shown on the left, and the size distribution is shown on the right. The sizes are indicated by different colors: 21 nt (blue), 22 nt (green), 23 nt (pink), and 24 nt (red).
(TIF)

S6 Fig Normalized abundance and distribution of small RNAs from four young *MIR* loci overproducing small RNAs in *35S*:*RTL1* plants. Small RNA abundance was normalized to the total amount of conserved miRNAs. The distribution of small RNAs along the chromosome is shown on the left, and the size distribution is shown on the right. The sizes are indicated by different colors: 21 nt (blue), 22 nt (green), 23 nt (pink), and 24 nt (red).
(TIF)

S7 Fig RTL1 triggers the formation of a novel 24 nt siRNA species from the 3’UTR of the *At3g18145* gene. **A**) The Arabidopsis Information Resource (TAIR) annotation of the genomic region spanning the *At3g18145* gene. **B**) Predicted hairpin structure of the 3’ UTR of the *At3g18145* RNA. **C**) Distribution of small RNAs along the *At3g18145* locus in wild-type Col, *35S*:*RTL1*, and *dcl234*. Reads are normalized to the total number of conserved miRNA reads. **D**) Size distribution of small RNAs from the *At3g18145* locus in Col, *35S*:*RTL1*, and *dcl234* triple mutant. The number of reads of each size of small RNAs is indicated by a color code: 21 nt (blue), 22 nt (green), 23 nt (pink), and 24 nt (red). Reads are normalized to the total of conserved miRNAs. **E**) RNA gel blot detection of 24 nt siRNA from the 3’UTR of *At3g18145* in wild-type (Col), Col transformed with the *35S*:*RTL1* construct (*Col/RTL1*), and *dcl2dcl3dcl4* mutants transformed with the *35S*:*RTL1* construct (*dcl234/RTL1*). Transformants exhibiting the strongest RTL1 developmental phenotype were analyzed. LMW RNAs were hybridized with a probe complementary to the 24 nt siRNA from the 3’UTR of *At3g18145* and with *U6* as loading control.
(TIF)

S8 Fig Normalized abundance and distribution of protein-coding and non–protein-coding genes overproducing small RNAs in *35S*:*RTL1* plants. Small RNA abundance was normalized to the total amount of conserved miRNAs. The distribution of small RNAs along the chromosome is shown on the left, and the size distribution is shown on the right. The sizes are indicated by different colors: 21 nt (blue), 22 nt (green), 23 nt (pink), and 24 nt (red).
(TIF)

S9 Fig Effect of tagged-*RTL1* expression on development and PTGS. **A**) Representative phenotypes of wild-type plants (Col) and transgenic plants expressing the indicated tagged-*RTL1* constructs. **B**) Total RNA was extracted from two independent transformants expressing each of the indicated tagged-*RTL1* constructs and hybridized with a *TAS2* probe and with *U6* as a loading control. **C**) The same constructs were introduced into line *L1*. Total RNA was extracted from two independent transformants expressing each of the indicated tagged-*RTL1* constructs and hybridized with a *GUS* probe and with *U6* as a loading control. Note that the images of Fig 5C are internal to the images of this panel.
(TIF)

S10 Fig RNA gel blot analysis of artificial miRNAs against RTL1. **A**) RNA gel blot detection of the artificial miRNA amiR-RTL1b in *N*. *benthamiana* leaves infiltrated with constructs under the control of the 35S (*35S*::*amiRb*) or UBQ10 (*UBQ10*::*amiRb*) promoter. Both constructs produced similar amounts of amiR-RTL1b. **B**) RNA gel blot detection of the artificial miRNAs amiR-RTL1a and amiR-RTL1b in a series of transgenic *Arabidopsis* carrying the *UBQ10*::*amiRa* or *UBQ10*::*amiRb* constructs.
(TIF)

S11 Fig Mutation in the RTL1 RNaseIII domain abolishes TYMV hypersusceptibility. Pictures of mock- and TYMV-inoculated wild-type (Col), *35S*:*RTL1-Myc* and *35S*:*RTL1mR3-Myc* plants. Ten-d-old plants were inoculated. Pictures were taken three weeks following inoculation.
(TIF)

S12 Fig Alignment of plant RTL1 amino acid sequences. Alignment of RTL1 amino acids sequences from *Arabidopsis thaliana* (NP_680697.4), *Brachypodium distachyon* (XP_003560753.1), *Cicer arietinum* (XP_004502137.1), *Cucumis sativus* (XP_004159229.1), Fragaria vesca (XP_004287969.1), *Oryza sativa* (NP_001057601.1), *Populus trichocarpa* (XP_002301611.1), *Solanum lycopersicum* (XP_004243532.1), *Sorghum bicolor* (XP_002444209.1), *Vitis vinifera* (XP_002270948.2), and *Zea mays* (ACG34313.1).
(TIF)

S1 Table Characteristics of small RNA libraries. All reads: total number of reads per library. 17–30: number of reads ranging between 17 and 30 nt. Mapped: number of 17–30 nt reads matching the 5 Arabidopsis chromosomes. No rRNA-tRNA: number of 17–30 nt reads matching the 5 Arabidopsis chromosomes after eliminating rRNA and tRNA. Total miRNA: number of reads matching the hairpins of all registered miRNA. Conserved miRNA: number of reads corresponding to the mature form of the 27 conserved miRNAs
(XLSX)

S2 Table Nonoverlapping regions exhibiting a differential production of small RNA between Col and *35S*:*RTL1* plants. (XLS)

S3 Table Clustering of differential regions into loci. Maximal distance admitted to define a locus = 1 kb.
(XLS)

S4 Table Oligonucleotide sequences. (DOC)

S1 Text Description of the 13 loci overaccumulating small RNA accumulation in 35S:RTL1 plants. (DOCX)

## Abbreviations

AGO: argonaute
CMV: cucumber mosaic virus
DCL: Dicer-like
*dcl234*: *dcl2 dcl3 dcl4* triple mutant
DRB: dsRNA binding domain
dsRBD: double stranded RNA binding domain
dsRNA: double stranded RNA
endoIR-siRNA: endogenous inverted repeat-derived siRNA
ETS: 3’External Transcribed Spacer
HMW: high molecular weight
LMW: low molecular weight
miRNA: microRNA
p4-siRNA: POLYMERASE IV-dependent siRNA
PTGS: post-transcriptional gene silencing
RDR: RNA-dependent RNA polymerase
RTL: RNASE THREE-LIKE
RT-PCR: reverse transcriptase polymerase chain reaction
siRNA: small interfering RNA
ta-siRNA: trans acting siRNA
TAIR: The Arabidopsis Information Resource
TCV: turnip crinkle virus
TVCV: turnip vein cleaning virus
TYMV: turnip yellow mosaic virus
VSR: viral suppressor of RNA silencing
