# Supplementary figures and images for: Plants Encode a General siRNA Suppressor That Is Induced and Suppressed by Viruses
Source: PLoS Biol. 2015 Dec 22;13(12):e1002326. doi: 10.1371/journal.pbio.1002326 (PMC4687873; doi:10.1371/journal.pbio.1002326)

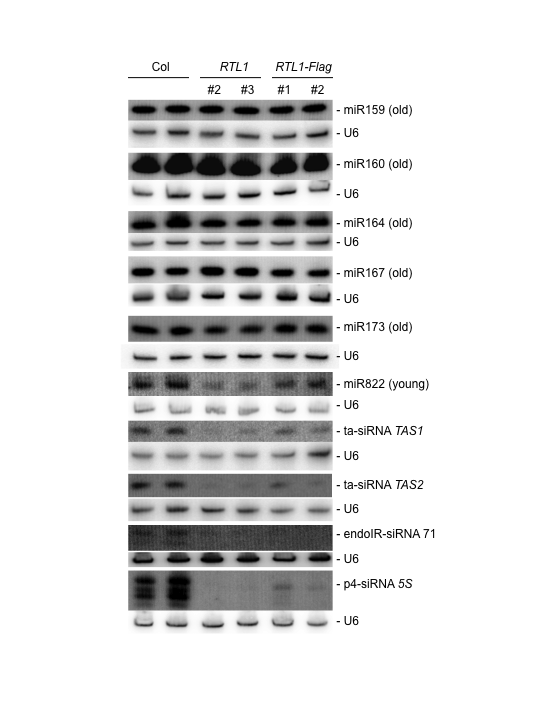

Supplement: S1 Fig — Ten identical gel blots of total RNA from flowers of wild-type (Col), 35S:RTL1 (RTL1) and 35S:RTL1-Flag (RTL1-Flag) plants were hybridized each with a different probe and then rehybridized with U6 as a loading control. A simplified figure showing one representative U6 control is shown in Fig 2C. (TIF) [file pbio.1002326.s002.tif]

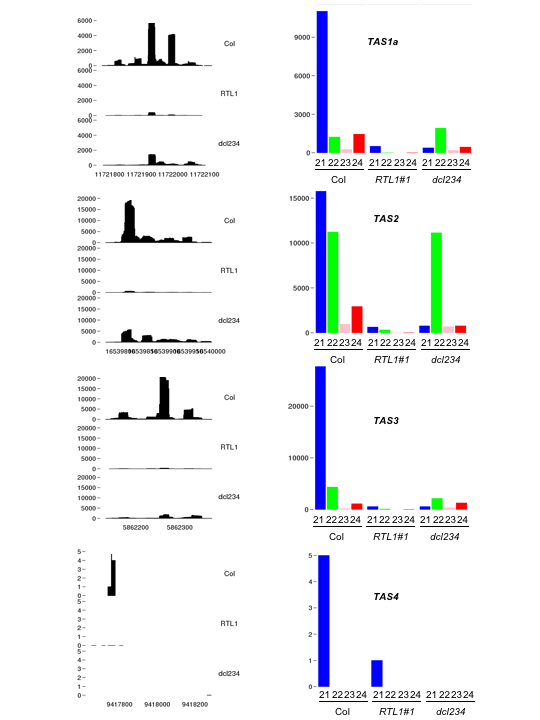

Supplement: S2 Fig — Small RNA abundance was normalized to the total amount of conserved miRNAs. The distribution of ta-siRNAs along the chromosome is shown on the left, and the size distribution is shown on the right. The sizes are indicated by different colors: 21 nt (blue), 22 nt (green), 23 nt (pink), and 24 nt (red). (TIF) [file pbio.1002326.s003.tif]

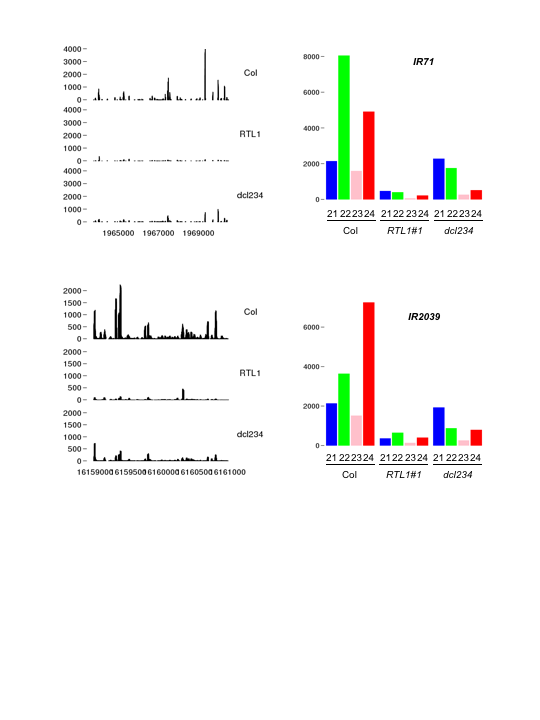

Supplement: S3 Fig — Small RNA abundance was normalized to the total amount of conserved miRNAs. The distribution of endoIR-siRNAs along the chromosome is shown on the left, and the size distribution is shown on the right. The sizes are indicated by different colors: 21 nt (blue), 22 nt (green), 23 nt (pink), and 24 nt (red). (TIF) [file pbio.1002326.s004.tif]

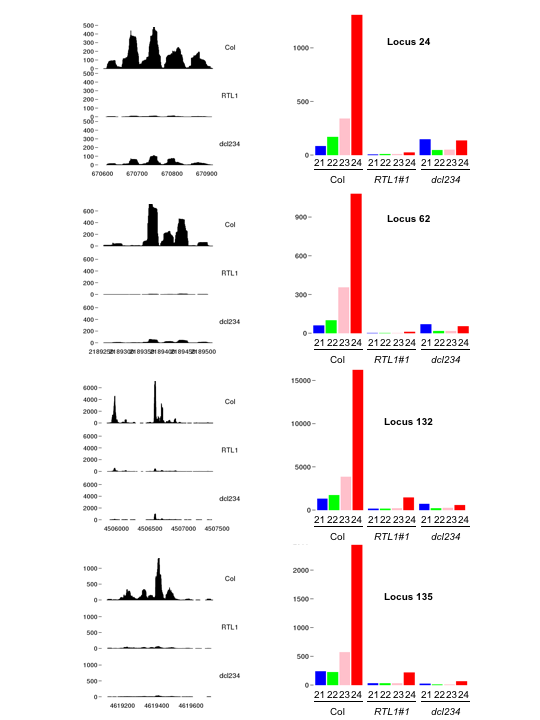

Supplement: S4 Fig — Small RNA abundance was normalized to the total amount of conserved miRNAs. The distribution of PolIV/PolV-siRNAs along the chromosome is shown on the left, and the size distribution is shown on the right. The sizes are indicated by different colors: 21 nt (blue), 22 nt (green), 23 nt (pink), and 24 nt (red). (TIF) [file pbio.1002326.s005.tif]

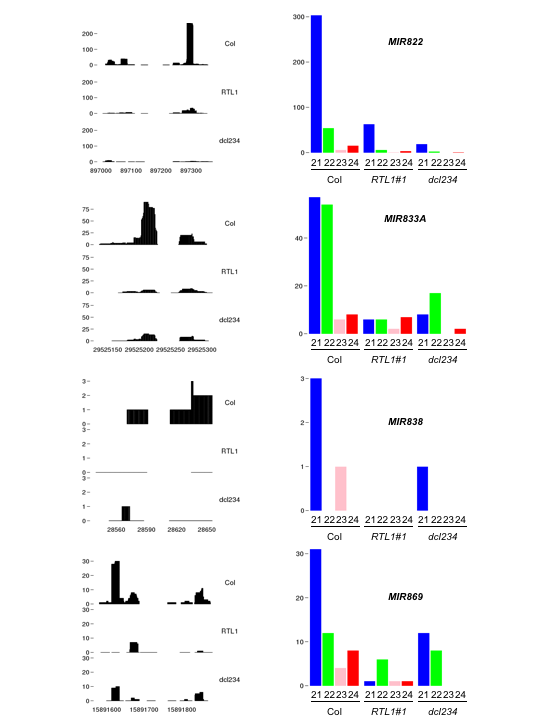

Supplement: S5 Fig — Small RNA abundance was normalized to the total amount of conserved miRNAs. The distribution of young miRNAs along the chromosome is shown on the left, and the size distribution is shown on the right. The sizes are indicated by different colors: 21 nt (blue), 22 nt (green), 23 nt (pink), and 24 nt (red). (TIF) [file pbio.1002326.s006.tif]

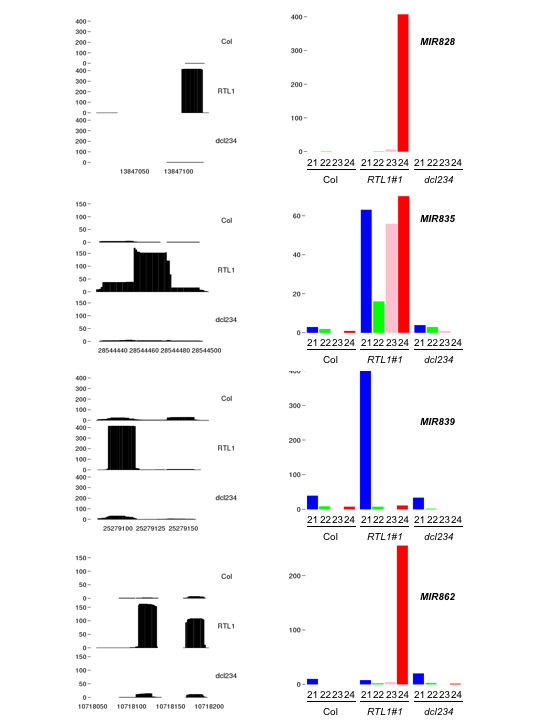

Supplement: S6 Fig — Small RNA abundance was normalized to the total amount of conserved miRNAs. The distribution of small RNAs along the chromosome is shown on the left, and the size distribution is shown on the right. The sizes are indicated by different colors: 21 nt (blue), 22 nt (green), 23 nt (pink), and 24 nt (red). (TIF) [file pbio.1002326.s007.tif]

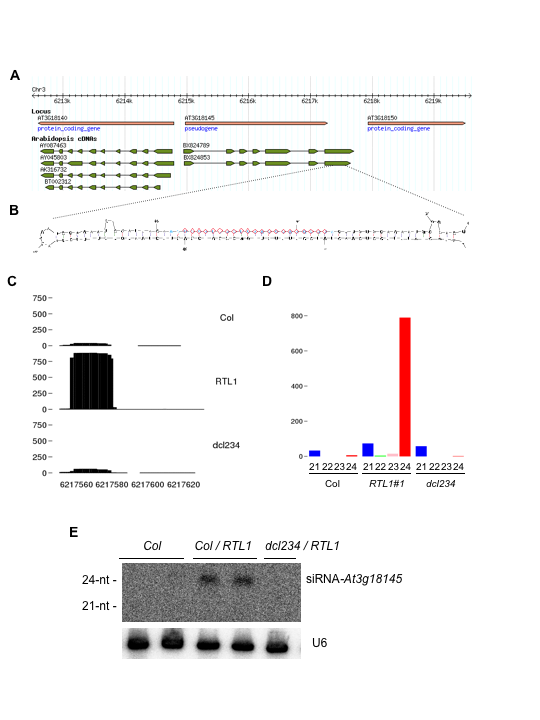

Supplement: S7 Fig — A) The Arabidopsis Information Resource (TAIR) annotation of the genomic region spanning the At3g18145 gene. B) Predicted hairpin structure of the 3’ UTR of the At3g18145 RNA. C) Distribution of small RNAs along the At3g18145 locus in wild-type Col, 35S:RTL1, and dcl234. Reads are normalized to the total number of conserved miRNA reads. D) Size distribution of small RNAs from the At3g18145 locus in Col, 35S:RTL1, and dcl234 triple mutant. The number of reads of each size of small RNAs is indicated by a color code: 21 nt (blue), 22 nt (green), 23 nt (pink), and 24 nt (red). Reads are normalized to the total of conserved miRNAs. E) RNA gel blot detection of 24 nt siRNA from the 3’UTR of At3g18145 in wild-type (Col), Col transformed with the 35S:RTL1 construct (Col/RTL1), and dcl2dcl3dcl4 mutants transformed with the 35S:RTL1 construct (dcl234/RTL1). Transformants exhibiting the strongest RTL1 developmental phenotype were analyzed. LMW RNAs were hybridized with a probe complementary to the 24 nt siRNA from the 3’UTR of At3g18145 and with U6 as loading control. (TIF) [file pbio.1002326.s008.tif]

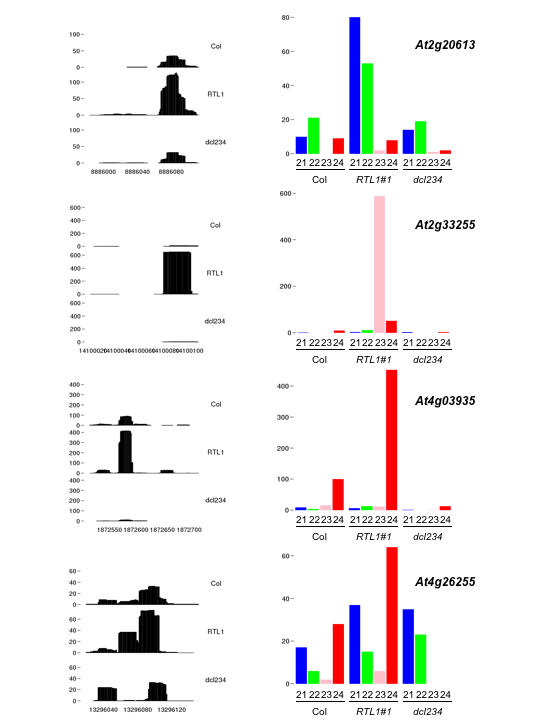

Supplement: S8 Fig — Small RNA abundance was normalized to the total amount of conserved miRNAs. The distribution of small RNAs along the chromosome is shown on the left, and the size distribution is shown on the right. The sizes are indicated by different colors: 21 nt (blue), 22 nt (green), 23 nt (pink), and 24 nt (red). (TIF) [file pbio.1002326.s009.tif]

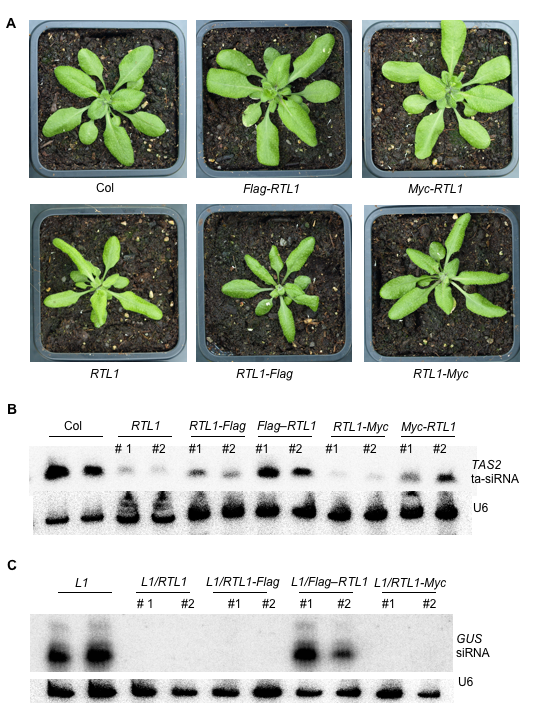

Supplement: S9 Fig — A) Representative phenotypes of wild-type plants (Col) and transgenic plants expressing the indicated tagged-RTL1 constructs. B) Total RNA was extracted from two independent transformants expressing each of the indicated tagged-RTL1 constructs and hybridized with a TAS2 probe and with U6 as a loading control. C) The same constructs were introduced into line L1. Total RNA was extracted from two independent transformants expressing each of the indicated tagged-RTL1 constructs and hybridized with a GUS probe and with U6 as a loading control. Note that the images of Fig 5C are internal to the images of this panel. (TIF) [file pbio.1002326.s010.tif]

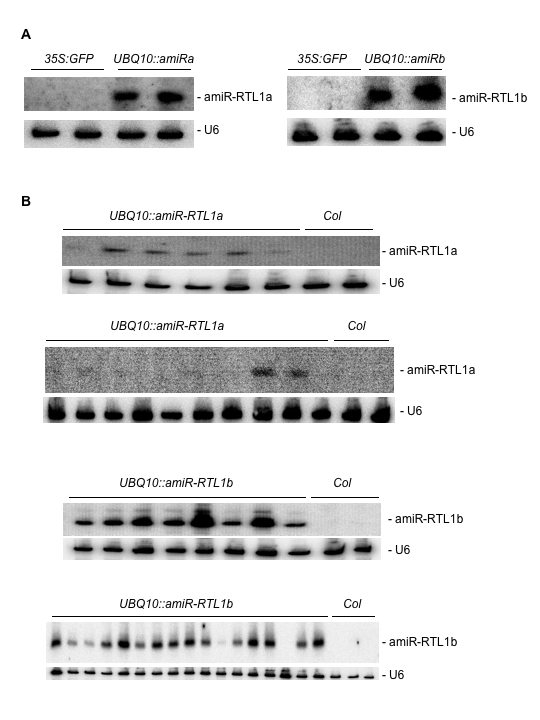

Supplement: S10 Fig — A) RNA gel blot detection of the artificial miRNA amiR-RTL1b in N. benthamiana leaves infiltrated with constructs under the control of the 35S (35S::amiRb) or UBQ10 (UBQ10::amiRb) promoter. Both constructs produced similar amounts of amiR-RTL1b. B) RNA gel blot detection of the artificial miRNAs amiR-RTL1a and amiR-RTL1b in a series of transgenic Arabidopsis carrying the UBQ10::amiRa or UBQ10::amiRb constructs. (TIF) [file pbio.1002326.s011.tif]

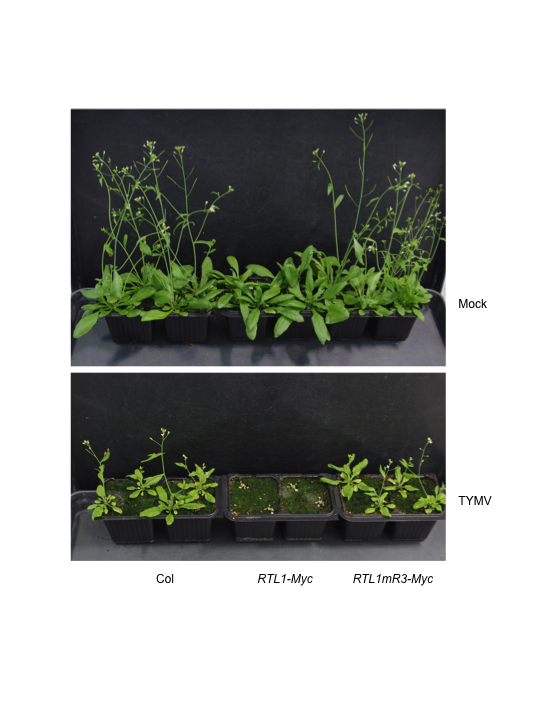

Supplement: S11 Fig — Pictures of mock- and TYMV-inoculated wild-type (Col), 35S:RTL1-Myc and 35S:RTL1mR3-Myc plants. Ten-d-old plants were inoculated. Pictures were taken three weeks following inoculation. (TIF) [file pbio.1002326.s012.tif]

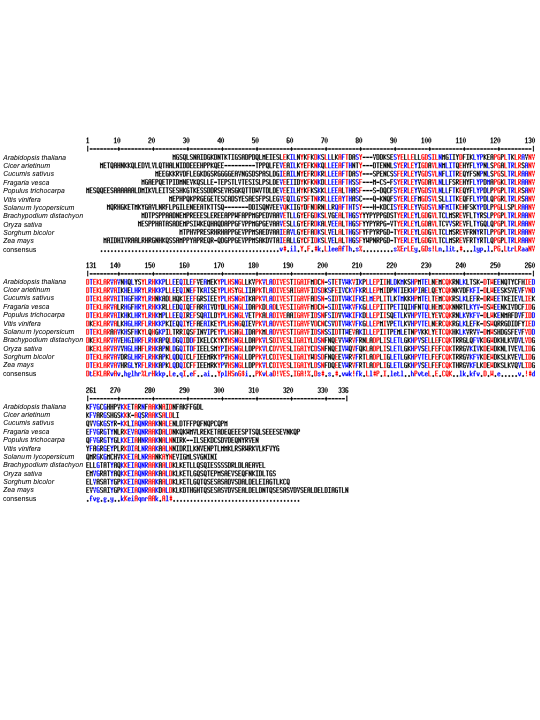

Supplement: S12 Fig — Alignment of RTL1 amino acids sequences from Arabidopsis thaliana (NP_680697.4), Brachypodium distachyon (XP_003560753.1), Cicer arietinum (XP_004502137.1), Cucumis sativus (XP_004159229.1), Fragaria vesca (XP_004287969.1), Oryza sativa (NP_001057601.1), Populus trichocarpa (XP_002301611.1), Solanum lycopersicum (XP_004243532.1), Sorghum bicolor (XP_002444209.1), Vitis vinifera (XP_002270948.2), and Zea mays (ACG34313.1). (TIF) [file pbio.1002326.s013.tif]
