# Supplementary material for: Plants Encode a General siRNA Suppressor That Is Induced and Suppressed by Viruses
Source: PLoS Biol. 2015 Dec 22;13(12):e1002326. doi: 10.1371/journal.pbio.1002326 (PMC4687873; doi:10.1371/journal.pbio.1002326)
Supplement: S4 Table — (DOC) [file pbio.1002326.s017.doc]

| Name | DNA Sequence |
| --- | --- |
| Oligonucleotides used for RT and PCR amplification and mutagenesis | |
| GAPDH- F5 | GGTACGACAACGAATGGGGT |
| GAPDH- R5 | TGACTGCGCATGGAATCAGT |
| RTL3_RT_fw | GGAAGTTGTTTGAAATTTGCGCC |
| RTL3_RT_rev | TTCTGCTCGTTTTGTTGCCC |
| RTL2-3 | CTCAAGGCTCTAATCATGTG |
| RTL2-10 | GATATCGCAAGGCTGATCGCTGC |
| RTL1-6 | GTTCTGTCATTGGATGGCTC |
| RTL1-7 | CCAAAACCATCGGATCAGCGG |
| RTL1-qPCR-5R | CAACGGACCAGGTGCTTCTT |
| attB1RTL1F | GGGGACAAGTTTGTACAAAAAAGCAGGCTTGATGGGATCGCAACTCTCAAA |
| attB2RTL1R | GGGGACCACTTTGTACAAGAAAGCTGGGTAAAGGTCTCCGAAAAATTTAGC |
| attB2RTL1-R3-R | GGGGACCACTTTGTACAAGAAAGCTGGGTAGTCTAAATGTATTATTGGCTCCAAC |
| RTL1m2-F | GAATCGTACGCACTCCTAGAACTTTTAGGAGCTTCGATCC |
| RTL1m2-R | GGATCGAAGCTCCTAAAAGTTCTAGGAGTGCGTACGATTC |
| 5rtl1BspH1 | TTGTCATGACAAGGTCTCCGAAAAATTTAGC |
| 3rtl1BspH1 | CCTCATGACAAGGTCTCCGAAAAATTTAGCAAAATTGTC |
| 5rtl1NdeI | CATATGAACAAGACACAAACAC |
| 3rtl1XhoI | CTCGAGAAGGTCTCCGAAAAATTTAGC |
| 5e86 | CGTTGATGACAAGACTGCATCGTACGAACTCCTAG |
| 3e86 | CTAGGAGTTCGTACGATGCACTCTTGTCATCAACG |
| 5e92 | CGTACGAACTCCTATGGCTTTTAGGAGATTCG |
| 3e92 | CGAATCTCCTAAAAGCCATAGGAGTTCGTACG |
| 5d96 | CCTAGAACTTTTAGGAGCTTCGATCCTAAATATGG |
| 3d96 | CCATATTTAGGATCGAAGCTCCTAAAAGTTCTAGG |
| 5e89d96 | GAATCGTACGCACTCCTAGAACTTTTAGGAGCTTCGATCC |
| 3e89d96 | GGATCGAAGCTCCTAAAAGTTCTAGGAGTGCGTACGATTC |
| IR71fwd1 | AAATGACCGCTACACTGCTTATCT |
| IR71rev1 | TCTCTCGTCAATGGACAATGAATC |
| IR71fwd2 | TAGGAAAGCTCGAACCCTTGG |
| IR71rev2 | TGAATGGTAATGATTTGTCTC |
| IR71rt | GATATGAGGTGCTTTGAATGG |
| U3fwd | ACGGACCTTACTTGAACAGGATCTG |
| U3rt | CTGTCAGACCGCCGTGCGA |
| Oligonucleotides used as probes for small RNA detection | |
| miR159 | TAGAGCTCCCTTCAATCCAAA |
| miR160 | TGGCATACAGGGAGCCAGGCA |
| miR173 | GTGATTTCTCTCTGCAAGCGAA |
| miR167 | TAGATCATGCTGGCAGCTTCA |
| miR164 | TGCACGTGCCCTGCTTCTCCA |
| miR156 | GTGCTCACTCTCTTCTGTCA |
| miR839 | GGGAACGATGAAAGGTTGGTA |
| amiR-RTL1a | AAGAGTGAATCGTACGAACTA |
| amiR-RTL1b | CGGGTCCCTGTAAATCACCAA |
| TAS1 | TACGCTATGTTGGACTTAGAA |
| TAS2 | GTGCTTCACAATGCTCTTTC |
| siR1003 | ATGCCAAGTTTGGCCTCACGGTCT |
| siR02 | GTTGACCAGTCCGCCAGCCGAT |
| IR71 | TCCTTTCCCTTTCCCTTTCTAC |
| U6 | TTGCGTGTCATCCTTGCGCAGG |
