# Supplementary material for: Plants Encode a General siRNA Suppressor That Is Induced and Suppressed by Viruses
Source: PLoS Biol. 2015 Dec 22;13(12):e1002326. doi: 10.1371/journal.pbio.1002326 (PMC4687873; doi:10.1371/journal.pbio.1002326)
Supplement: S1 Text — (DOCX) [file pbio.1002326.s018.docx]

**Description of the 13 loci over-accumulating small RNA accumulation in 35S:RTL1 plants**

The 13 loci for which small RNA accumulation was increased in *35S:RTL1* plants included three conserved MIR genes (*MIR171a, MIR397b, MIR408*), five non-conserved MIR genes (*MIR828, MIR835, MIR839, MIR862, MIR3440b*), two genes producing long non-coding RNAs (*At4g03935*, *At4g26255*), two protein-coding genes (*At2g20613*, *At3g33255*), and one transcribed non-annotated region adjacent to the centromer of chromosome 3. Given that conserved miRNAs are generally not affected by RTL1 (Figure 2C), increased transcription of *MIR171a, MIR397b* and *MIR408* genes in *35S:RTL1* plants due to the stressed nature of these plants could explain the increased miRNA accumulation. Consistent with this hypothesis, miR171, miR397 and miR408 accumulation was previously shown to respond to various stresses [52,53,54,55]. At four non-conserved MIR loci (*MIR828, MIR835, MIR862, MIR3440b*), the most abundant small RNA found in *35S:RTL1* does not correspond to the canonical 21-nt miRNA found in Col and instead is a 24-nt species (Supplemental Figures S5 and S6). To explain these results, we propose that in most cases (~6000 loci), RTL1 cleaves the long dsRNA before it is processed by the DCLs, and that the resulting fragments are subsequently degraded by 5’-to-3’ and/or 3’-to-5’ exoribonucleases. In very rare cases (5 young MIR genes), RTL1-mediated cleavage produces stable dsRNA molecules that can be processed by the regular DCL (*MIR839*) or a different DCL (*MIR828, MIR835, MIR862, MIR3440b*), leading to the appearance of novel small RNA species. Supporting this hypothesis, the accumulation of the novel 24-nt species derived from *MIR3440b/At3g18145* was abolished when the *35S:RTL1* construct was introduced into the *dcl2dcl3dcl4* triple mutant (Supplemental Figure S6), indicating that this 24-nt species is not a direct product of RTL1, but rather results from the processing of the hairpin by DCL3 following primary cleavage by RTL1 (see in vitro assay later on). Changes in the small RNA profile were also observed when analyzing the protein-coding genes *At3g33255* and *At2g20613* (Supplemental Figure S7). Indeed, a novel 23-nt species is produced by the fourth intron of *At3g33255* in *35S:RTL1* plants, while the ratio between 21- and 22-nt produced from *At2g20613* is modified in *35S:RTL1* plants. In contrast, no change in the small RNA profile was observed when analyzing the non-protein coding genes *At4g03935* and *At4g26255* (Supplemental Figure S7), suggesting that in these two cases RTL1-mediated cleavage of the dsRNA precursor does not change the specificity towards the DCLs, but simply stabilizes the precursor, thus promoting its processing by the regular DCLs. Together, these results strongly suggest that in most cases, RTL1-mediated cleavage of dsRNA precursors prevents their processing by the DCLs, but that in very rare cases, RTL1-mediated cleavage stabilizes the precursor, thus promoting its processing by regular or alternative DCLs.
